# Macrophage Subpopulation Promotes Skeletal Muscle Regeneration Through HGF/MET Signaling‐Mediated Skeletal Muscle Stem Cell Proliferation

**DOI:** 10.1111/acel.70042

**Published:** 2025-03-25

**Authors:** Hiroyuki Koike, Miho Sugimura, Rie Ouchi, Yuki Yoshimoto, Ichiro Manabe, Yumiko Oishi

**Affiliations:** ^1^ Department of Medical Biochemistry Graduate School of Medical and Dental Sciences, Institute of Science Tokyo Tokyo Japan; ^2^ Professional Development Center Institute of Science Tokyo Hospital Tokyo Japan; ^3^ Department of Molecular Craniofacial Embryology and Oral Histology Graduate School of Medical and Dental Sciences, Institute of Science Tokyo Tokyo Japan; ^4^ Department of Systems Medicine Chiba University Graduate School of Medicine Chiba Japan

**Keywords:** macrophage, organoid, regeneration, single‐cell RNA‐sequence, skeletal muscle stem cells

## Abstract

Macrophages are key regulators of skeletal muscle regeneration, yet the specific macrophage subpopulations responsible for this process and their cell–cell interactions remain insufficiently understood, as does the mechanism underlying age‐related impairment of skeletal muscle regeneration. We utilized single‐cell RNA sequencing to identify transcriptionally distinct macrophage subpopulations within skeletal muscle from young (8‐week‐old) and aged (24‐month‐old) mice. Among them, the Mac_1 subpopulation interacted with muscle satellite cells (MuSCs) and promoted their proliferation through HGF/c‐Met signaling that suppressed *Cdkn1b* expression. This interaction was critical for efficient muscle regeneration in vivo and in a 3D‐muscle organoid model. The age‐related decline in muscle regeneration was associated with reduced HGF expression in Mac_1 macrophages. Administration of exogenous HGF to aged mice and macrophage‐depleted young mice partially rescued the impaired muscle regeneration. This study elucidates a mechanism of skeletal muscle regeneration that offers insight into potential strategies for preventing and treating skeletal muscle diseases, including sarcopenia.

## Graphical abstract

This study identifies a macrophage subpopulation, Mac_1, that promotes skeletal muscle regeneration by enhancing muscle stem cell proliferation through HGF/MET signaling. A 3D‐muscle organoid model confirmed the critical role of HGF/MET signaling in regeneration, highlighting a potential therapeutic target for age‐related impairment of skeletal muscle regeneration.

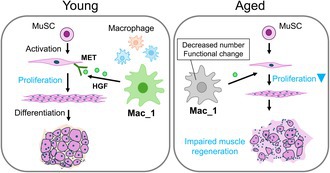

## 1 Introduction

Skeletal muscle functions as an organ of locomotion and regulates metabolism as the largest energy‐consuming organ. The quality and quantity of skeletal muscle are known to decline significantly with age, a condition known as sarcopenia. The onset and progression of sarcopenia can lead to complications such as diabetes and dyslipidemia as well as frailty, which can significantly reduce the quality of life in the elderly. Therefore, with the aging of populations around the world, there is an urgent need to establish a treatment for sarcopenia, and that requires elucidating the molecular mechanisms involved in sarcopenia, the details of which are still not fully understood.

Skeletal muscle tissue has very high regenerative capacity. Within skeletal muscle, minor damage with cell death is ongoing nearly constantly (e.g., during daily exercise), and regeneration and repair are repeated after each injury. This regeneration and repair reportedly decreases with age, which is considered one of the causes of the onset and progression of sarcopenia (Lo et al. 2020). The cells essential to the regeneration and repair process are skeletal muscle stem cells (MuSCs). During the regeneration process, MuSCs are stimulated to proliferate and differentiate into myofibers (Sousa‐Victor et al. 2022). In addition, various stromal cells, including macrophages, neutrophils, endothelial cells, and fibro‐adipose progenitor cells (FAPs), play supportive roles (Sousa‐Victor et al. 2022). Of these, macrophages contribute to the process of skeletal muscle regeneration by regulating the activation, proliferation, and differentiation of MuSCs and FAPs (Dort et al. 2019; Koike et al. 2022; Oishi and Manabe 2018; Wang et al. 2024). Consequently, determining the mechanisms underlying macrophage‐driven skeletal muscle regeneration is very important for the development of preventive and therapeutic approaches to sarcopenia.

Macrophages are known to be a highly heterogeneous cell type and to include tissue‐resident populations (Epelman et al. 2014; Wynn et al. 2013), among which are macrophages residing within skeletal muscle (Chakarov et al. 2019; Dick et al. 2022; Jaitin et al. 2019; Wang et al. 2020). Indeed, recent single‐cell transcriptome analyses revealed the presence of 11 distinct macrophage subpopulations within homeostatically intact skeletal muscle (Krasniewski et al. 2022), and several subpopulations with highly diverse functions are present at various times within regenerating muscle (de Micheli et al. 2020; Oprescu et al. 2020). Notably, the diversity of macrophage subpopulations and their functions within homeostatic skeletal muscle change with age (Krasniewski et al. 2022). This suggests that age‐related changes in macrophages may reduce the regenerative capacity of skeletal muscle by reducing the coordination among MuSCs and other cell types, leading to the onset and progression of sarcopenia. Given that macrophages generate more diverse subpopulations during skeletal muscle regeneration and cooperate with MuSCs and other cells to regulate skeletal muscle regeneration, changes in macrophages during the process are assumed to impact muscle regeneration significantly. However, the age‐related changes in macrophages during skeletal muscle regeneration and the mechanisms underlying the diminished coordination between the macrophages and MuSCs involved in sarcopenia remain largely unknown.

To address the age‐related changes in macrophages during skeletal muscle regeneration, we analyzed the dynamic interactions between macrophages and MuSCs that contribute to the decline in regenerative capacity with age. By combining single‐cell transcriptome analysis with a newly developed three‐dimensional muscle organoid system, we discovered that specific macrophage subsets promote MuSC proliferation by suppressing the expression of the cell growth inhibitor Cdkn1b via HGF/c‐MET signaling. Additionally, we found that HGF expression is downregulated in macrophages within regenerating muscle in aged mice. Administering HGF to mice lacking macrophages or to aged mice restored skeletal muscle regeneration. These findings suggest that specific macrophage subpopulations exhibiting induced HGF/c‐MET signaling are crucial for maintaining muscle regenerative capacity, particularly in the context of aging.

## 2 Results

### 2.1 Aging Delays Skeletal Muscle Regeneration and Repair After Injury

Skeletal muscle regeneration in response to local tissue injury is a complex and dynamic process coordinated by the interaction of multiple cell types. To investigate muscle regeneration, we used a model in which the regeneration process was studied following cardiotoxin (CTX)‐induced injury to the tibialis anterior (TA) muscles of wild‐type C57BL/6J mice (Lepper et al. 2009). Histological analysis of young (8‐week‐old) mice throughout the regeneration process showed that skeletal muscle tissue was severely damaged on the first day after CTX injection. By day 3, numerous immune cells had accumulated at sites of injury, and the injured muscle fibers were surrounded by CD11b^+^CCR2^+^ macrophages (Figure S1A). On day 7 post‐injury, the myofiber regeneration was nearly complete, as indicated by the presence of MYOZ1 (Yoshimoto et al. 2020)‐positive, mature myofibers (Figure S1B,C). The same injury model was then used to compare the muscle regeneration and repair process in young and aged (24‐month‐old) mice. Histological analysis of samples collected 7 days post‐injury confirmed that skeletal muscle regeneration was delayed with persistent fibrosis and F4/80^+^ macrophage infiltration in aged mice, which is consistent with an earlier report (Moiseeva et al. 2023) (Figure S2A–C).

### 2.2 scRNA‐Seq Reveals Age‐Related Changes in Transcriptional Expression During Muscle Regeneration

**FIGURE 1:**
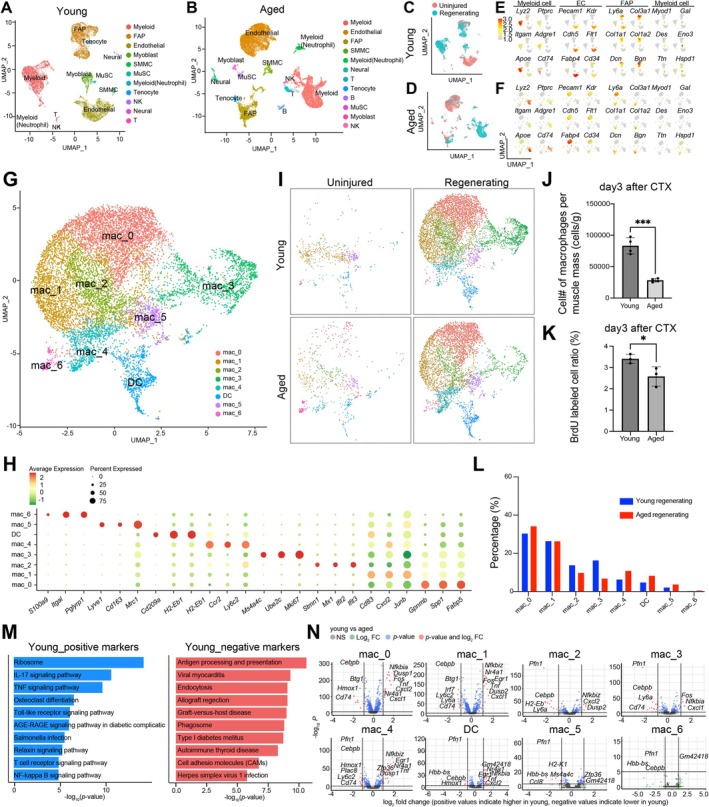
Aging impacts the proportion and characteristics of the seven subpopulations of macrophages in regenerating skeletal muscle tissue. (A) Uniform Manifold Approximation and Projection (UMAP) plot of single‐cell transcriptomic data from young (8‐weeks‐old) mouse tibialis anterior (TA) muscle cells. Cells are colored based on major cell lineages. (B) UMAP plot of single‐cell transcriptomic data from aged (24‐month‐old) mouse TA muscle cells. Cells are colored based on major cell lineages. (C) UMAP plot with red indicates uninjured young TA muscle cells; blue indicates regenerating young TA muscle cells on day 3 after cardiotoxin injection. (D) UMAP plot with red indicates uninjured aged TA muscle cells; blue indicates regenerating aged TA muscle cells on day 3 after CTX injection. (E) UMAP plots of young TA muscle cells projecting the normalized expression of major marker genes for myeloid cells, endothelial cells, FAPs, and myogenic cells. (F) UMAP plots of aged TA muscle cells projecting the normalized expression of major marker genes for myeloid cells, endothelial cells, FAPs, and myogenic cells. (G) UMAP plot of single‐cell transcriptomic data from young and aged mouse macrophage and dendritic cell (DC) populations in TA muscle cells. The plot revealed cellular heterogeneity, with a total of eight distinct subclusters of macrophages and DCs. (H) Dot plots showing the expression of genes of interest across the DCs and seven macrophage subclusters for cluster identification. (I) UMAP projections of macrophages from young uninjured, young injured, aged uninjured, and aged injured muscles. Each dot is colored according to the macrophage subclusters in (G). (J) Cell numbers of young and aged macrophages per TA muscle mass on day 3 after CTX injection. (K) BrdU‐labeled cell ratio in young and aged macrophages (CD11b^+^/F4/80^+^) from TA muscles on day 3 after CTX injection. (L) The proportion of each macrophage subpopulation in scRNA‐seq data from uninjured skeletal muscle cells and regenerating skeletal muscles (day 3 after CTX injection) in young and aged mice. (M) The *p*‐values and names of the most overrepresented KEGG pathways were calculated based on all the genes differentially expressed between macrophages in young and aged regenerating skeletal muscles. Blue indicates the genes with increased expression in the young; red indicates the genes with increased expression in the aged. (N) Volcano plot comparing transcript levels between all cells within each macrophage subpopulation. log_2_ fold‐changes in normalized gene expression are plotted against −log_10_ adjusted *p*‐value. Positive log_2_ fold‐changes indicate higher expression in young mice, and negative log_2_ fold‐changes indicate lower expression in young mice. Differentially expressed genes (adjusted *p*‐value < 0.05) are colored light blue. Genes with a |log_2_ fold‐change| > 1 are labeled and colored pink.

To assess the changes in cell kinetics and interactions during skeletal muscle regeneration that occur with aging, on day 3 after injury, we collected single‐cell suspensions from the skeletal muscles of uninjured and injured young and aged mice for scRNA‐seq analysis. Day 3 post‐injury was chosen because that is when MyoD‐positive activated MuSCs were most abundant (Figure S1B,C). After quality control, 7770/10,900 cells were obtained from uninjured/injured tissues from young mice, while 11,339/7338 cells were collected from uninjured/injured tissues from aged mice (Figure 1A–D). Based on the expression of differentially expressed marker genes, the clusters were identified as myeloid cells, fibro‐adipose progenitor cells (FAPs), endothelial cells, myoblasts, smooth muscle cells (SMCs), MuSCs, neutrophils, tendon cells, NK cells, neurons, and T lymphocytes, among other cell types (Figure 1A–F).

### 2.3 Diverse Macrophage Subpopulations Were Present in the Injured Muscle Tissue of Young and Aged Mice

To understand the functional dynamics of macrophages during muscle regeneration, we analyzed the myeloid cell clusters in detail. After extracting only myeloid cell clusters from the four datasets (data from uninjured and injured muscle obtained from young and aged mice), unsupervised clustering yielded eight myeloid cell clusters: Mac_0 to Mac_6 and dendritic cells (DCs) (Figure 1G–I). Mac_4 expressed markers of pro‐inflammatory macrophages, such as *Ccr2* and *Ly6C2*, while Mac_5 expressed markers of anti‐inflammatory macrophages, including *Mrc1, Cd163*, and *Lyve1* (Figure 1H). The other clusters displayed unique and characteristic gene expression profiles—i.e., Mac_0 (described as Il7r + macrophage in previous report (Oprescu et al. 2020)) expressed *Gpnmb, Spp1*, and *Fabp5*; Mac_1 expressed *Cd83, Cxcl2*, and *Junb*; Mac_2 expressed *Mx1, Ifit2*, and *Ifit3*; Mac_3 (proliferating macrophages) expressed *Mki67* and *Ube2c*; DCs expressed *Cd209a, H2‐Eb1*, and *H2‐Ab1*; and Mac_6 expressed *S100a9, Itgal*, and *Pglyrp1* (Figure 1H).

### 2.4 Local Macrophage Proliferation in Response to Muscle Injury Declined With Age

In both young and aged mice, macrophage numbers dynamically increased within regenerating skeletal muscle treated with CTX (Figure 1I). In aged mice, however, there were significantly fewer CD45^+^/CD11b^+^/Ly6G^−^ macrophages present on day 3 post‐injury than in young mice (Figure 1J). In particular, the proportion of macrophages in the Mac_3 proliferative subpopulation was smaller in aged mice (Figure 1I). To confirm this observation, 3 days after injury, proliferating cells were labeled by peritoneally injecting BrdU. 1 h after the BrdU injection, cells were collected from the injured tissue, and BrdU‐positive cells were analyzed by flow cytometry, which revealed that the fraction of BrdU‐positive CD45^+^/CD11b^+^/Ly6G^−^ cells was significantly smaller in aged mice (Figure 1K). This suggests that age‐related reductions in a local macrophage proliferation contribute to the age‐related decline in macrophage numbers.

Differential gene expression (DGE) analysis of macrophages in young and aged mice at day 3 after CTX injection showed that the expression of inflammatory cytokine‐related gene sets, such as those related to ribosome, IL‐17 signaling, and TNF signaling, was reduced in aged mice, whereas antigen processing and presentation, viral myocarditis, and endocytosis sets were activated in aged mice (Figure 1M). DGE analysis of each macrophage subpopulation in young and aged mice showed that, with aging, expression of *Tnf* and *Nr4a1* was reduced, while *Cebpb* expression was activated in some macrophage subpopulations, including Mac_0, Mac_1, Mac_4, and DCs (Figure 1N). The expression of *Pfn1* increased with age in Mac_2, Mac_3, Mac_4, Mac_5, Mac_6, and DC, as did *Cd74* in Mac_0, Mac_1, Mac_3, Mac_4, and a wide range of subtypes, which suggests that the expression of these genes strongly correlates with age (Figure 1N). Thus, aging may affect macrophages' function during skeletal muscle regeneration by altering gene expression in the various macrophage subpopulations.

### 2.5 Identification of the Macrophage Subpopulation Mac_1 That Interacts With MuSCs

Previous studies suggested that macrophages interact with MuSCs during differentiation (Arnold et al. 2007; Fang et al. 2020). Because multiple macrophage populations with diverse functions have assembled within the injured tissue by 3 days post‐injury, we hypothesized that a specific population of macrophage subtypes may interact with MuSCs.

**FIGURE 2:**
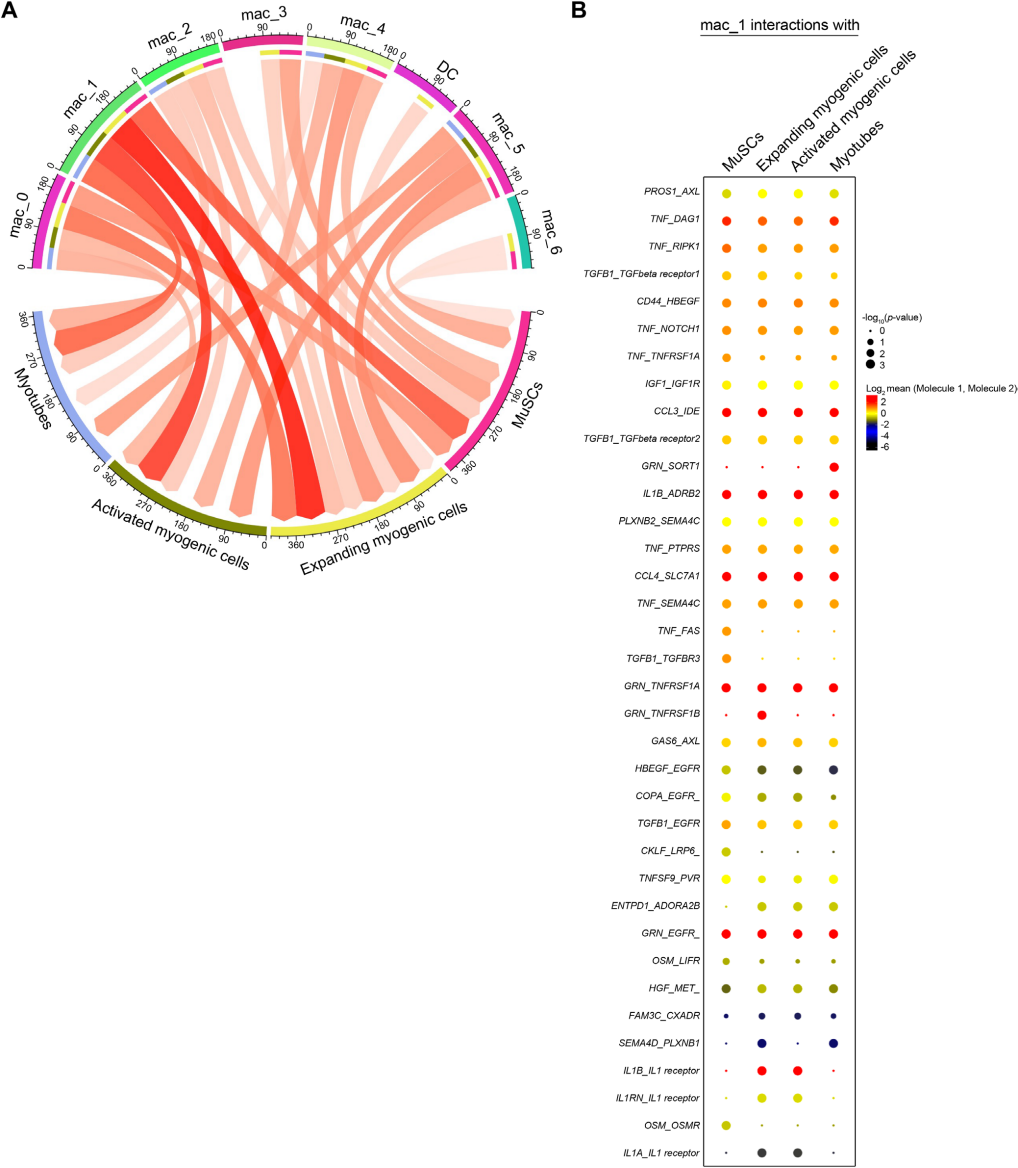
Strong ligand‐receptor interactions between Mac_1 and expanding MuSCs. (A) Ligand–receptor interactions between macrophage subpopulations and myogenic subtypes. Circos plot of the number of ligand–receptor interactions across all combinations from macrophages to myogenic subpopulations. The combination of subpopulations with the highest number of interactions has a darker shade of red (more transparency means less interaction). (B) A list of ligand receptors between Mac_1 and myogenic subtypes. CellPhoneDB makes a pairwise comparison between all cell types by randomly permuting labels of the clusters 1000 times (default) and determining the mean average expression levels of ligand receptors in the given interacting cluster pairs. The *p*‐values were generated by CellphoneDB, which uses a one‐sided permutation test to compute significant interactions. Each dot size shows the −log_10_ *p*‐value, and the color indicates the log_2_ mean of the expression values for the listed ligand‐receptor pairs (*y*‐axis) in the respective interacting cell types (*x*‐axis).

To identify the macrophage subpopulation(s) that interact explicitly with the myogenic cell population (including MuSCs and myoblasts in different states of differentiation that commonly express MyoD: Figure 1E,F), we combined scRNA‐seq data obtained from uninjured and injured muscle from young mice and performed ligand receptor analysis using the Cellphone DB platform (Figure 2A) (Efremova et al. 2020; Vento‐Tormo et al. 2018). In this analysis, myogenic cell populations were classified into four types: MuSCs (expressing *Pax7*, *Chodl* (Barruet et al. 2020; Saleh et al. 2022) and *Btg2* (de Micheli et al. 2020)), myotubes (expressing *Mymx*, *Myog*, and *Cdkn1c*) and activated myogenic cells (McKellar et al. 2021) (expressing *Myod1*) as well as a subcluster showing strong expression of proliferation‐related genes, including activated myogenic cells and expanding myogenic cells (expressing *Ube2c*, *Cdk1*, and *Mki67*). This classification enabled us to analyze macrophage‐MuSC interactions with respect to the differentiation stage. The results indicated that the Mac_1 subpopulation interacted more strongly with myogenic cell populations than did other macrophage subpopulations (Figure 2A). Notably, this included a strong interaction between Mac_1 and expanding MuSCs (Figure 2B and Figure S3).

To perform a Mac_1‐specific analysis, a search was carried out for surface antigens that would enable Mac_1 to be fractionated with flow cytometry. First, gene expression of membrane surface proteins used for flow cytometry (mainly CD antigens) was analyzed from single‐cell RNA sequencing analyses. This showed that Mac_0 and Mac_1 weakly expressed *Mrc1* (CD206) and *Ly6c2* (Ly6C) (Figure S4A). In addition, the expression of *Cd9* and *Gpnmb* was weaker in Mac_1 than in Mac_0. In other words, it was possible to fractionate Mac_1 by targeting the macrophage/DC population with weak expression of these surface antigens. We therefore used flow cytometry to fractionate cells weakly expressing CD9 and GPNMB from the myeloid population, excluding Ly6G‐positive neutrophils and the Ly6C‐ and CD206‐positive population (Figure S4B,C).

### 2.6 Mac_1 Interacts With MuSCs to Accelerate Proliferation Through HGF/MET Signaling

**FIGURE 3:**
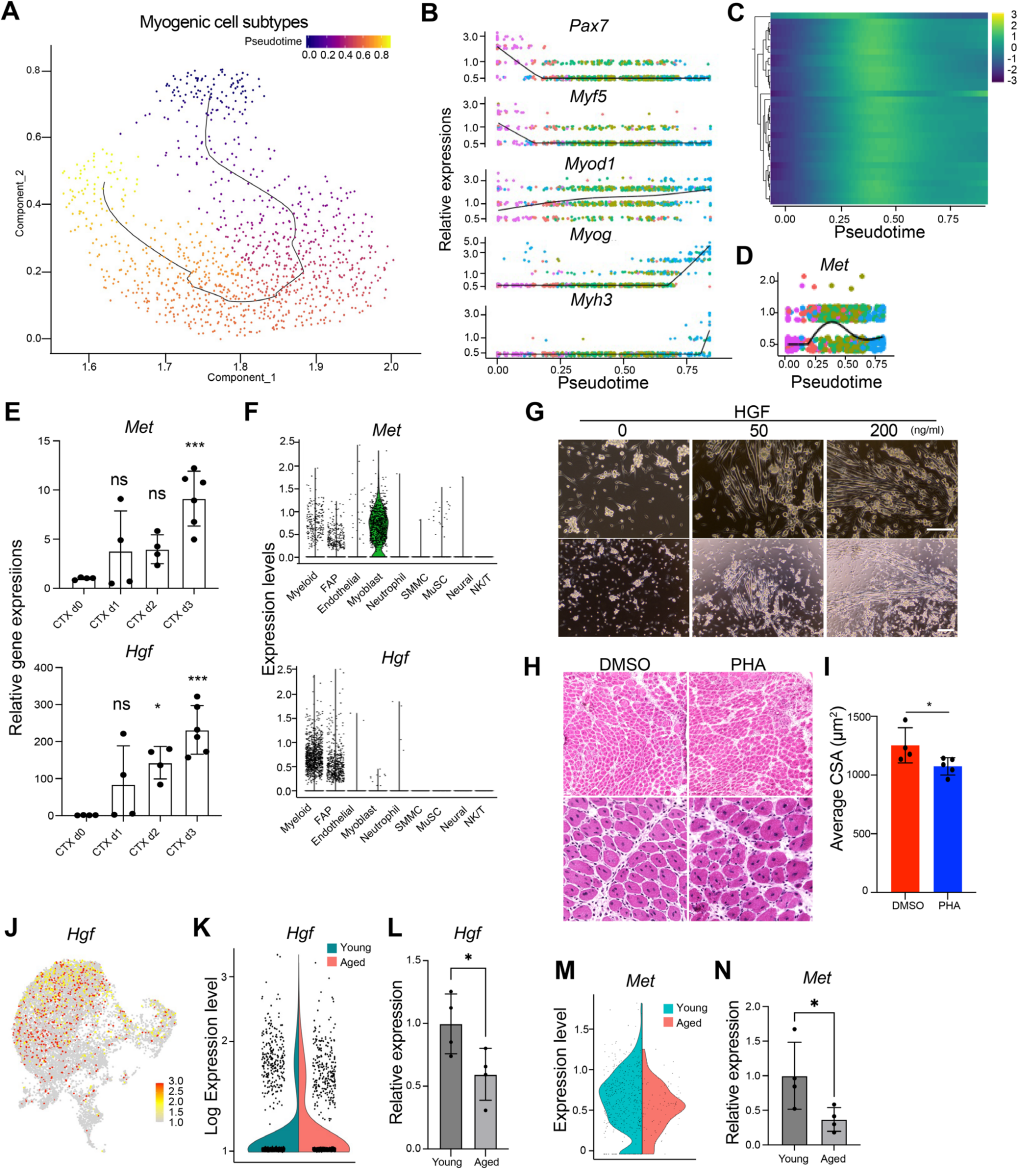
HGF/MET signaling promotes the proliferation of skeletal muscle stem cells in skeletal muscle regeneration. (A) Pseudo‐time trajectory of myogenic cell subtypes from skeletal muscle tissue. (B) Gene expression of *Pax7, Myf5, Myod1, Myog*, and *Myh3* across pseudo‐time. (C) Heatmap showing expression of G2/M‐phase marker genes across pseudo‐time. Higher expression is in yellow; lower expression is in dark blue. (D) Gene expression of *Met* across pseudo‐time. (E) Gene expression of *Met* and *Hgf* during skeletal muscle regeneration in CTX‐injected mice. *n* = 4. Data are expressed as the means ± SD. *p < 0.05 vs. CTX day 0 (one‐way ANOVA with Dunnett's multiple comparisons test). (F) Violin plot showing the expression of *Me*t and *Hgf* in each cell cluster. (G) Effect of HGF on the growth of skeletal muscle stem cells in vitro. Lower panels indicate the larger image of the upper panels. Scale: 200 μm for both upper and lower panels. (H) Effect of the c‐Met antagonist PHA‐665752 (PHA) on skeletal muscle regeneration in vivo. Scale: 100 μm. (I) Quantification of the cross‐sectional areas of the fibers in (G). *n* = 4 for DMSO and *n* = 5 for PHA. Data are expressed as the means ± SD. *p < 0.05 (unpaired two‐tailed Student's *t*‐test). (J) UMAP plots of young and aged macrophage/DC populations projecting the normalized expression of *Hgf* genes. (K) Violin plot of *Hgf* expression in the Mac_1 subpopulation. (L) qPCR analysis of *Hgf* expression in isolated CD45^+^/F4/80^+^ macrophages within regenerating skeletal muscle tissue from young and aged mice. *n* = 4 per group. Data are expressed as the means ± SD. *p < 0.05 (unpaired two‐tailed Student's *t*‐test). (M) Violin plot of *Met* expression in the myoblast subpopulation. (N) qPCR analysis of *Met* expression in isolated MuSCs from young and aged regenerating skeletal muscle. *n* = 4 per group. Data are expressed as the means ± SD. *p < 0.05 (unpaired two‐tailed Student's *t*‐test).

The observation that Mac_1 interacts with MuSCs during proliferation and differentiation suggests the expression of the receptor required for that interaction may be increased during that period. Monocle's pseudo‐time analysis of the myogenic cell population in muscle tissue from young mice showed the pseudo‐temporal trajectories from undifferentiated MuSCs to differentiated myotubes (Figure 3A–C). That result recapitulates the in vivo regeneration process, in which rapid downregulation of *Pax7* and *Myf5* is followed by upregulation of the myogenic regulatory factors *Myod1* and *Myog*. During that process, transient upregulation of cell cycle‐related genes was observed (Figure 3C). We next looked for receptors transiently induced in the expanding MuSCs. Among eight candidates (Figure S5), *Met* was strongly detected in MuSCs during the proliferation process (Figure 3D). C‐Met (encoded in the *Met* gene) is a receptor for HGF (Organ and Tsao 2011). The HGF/MET signal was also detected in the ligand‐receptor pairs connecting Mac_1 and all MuSC subtypes, with the highest correlation in expanding MuSCs (Figure 2B, Figure S3). This suggests that HGF/MET signaling may be necessary for promoting skeletal muscle regeneration mediated by the interaction between Mac_1 and MuSCs.

To confirm the scRNA‐seq data, total RNA was independently obtained from mouse muscle tissue during regeneration after injury, and mRNA expression was analyzed. *Met* and *Hgf* expression was induced in the injured tissue 3 days post‐injury (Figure 3E), which supports the idea that HGF/MET signaling is upregulated after muscle injury. In addition, *Met* was exclusively expressed in the myogenic cell population (Figure 3F), while *Hgf* expression was detected in both macrophages and FAPs. Because large numbers of macrophages had accumulated within the muscle tissue by day 3 post‐injury, the contribution of macrophages to the secretion of *Hgf* was considered significant (Figure 3F). We then investigated the effect of HGF in MuSCs in vitro. Consistent with past findings (Miller et al. 2000), HGF administration dose‐dependently induced the proliferation of primary mouse MuSCs (Figure 3G). Treating mice undergoing skeletal muscle regeneration with PHA‐665752, a MET inhibitor, significantly reduced the diameters of regenerated skeletal muscle fibers measured on day 7 (Figure 3H,I). These findings suggest that the HGF/MET signaling pathway may be critical to Mac_1‐mediated proliferation of MuSCs during skeletal muscle regeneration. Moreover, we found that *Hgf* expression was weaker in macrophages within regenerating skeletal muscle tissue from aged mice than young mice (Figure 3J–L), which suggests that reduced Mac_1‐derived HGF expression could be responsible for the failure of skeletal muscle regeneration in aged mice. Furthermore, mRNA expression analysis of isolated MuSCs confirmed a reduction in *Met* expression in MuSCs in regenerating skeletal muscle tissue from aged mice compared to young mice, consistent with our scRNA‐seq findings (Figure 3M,N). Taken together, these findings demonstrate that specific macrophage subsets interact with MuSCs via HGF/MET signaling to promote their proliferation during skeletal muscle regeneration.

### 2.7 Muscle Organoids Reproduce Muscle Differentiation Through Interaction With Macrophages

**FIGURE 4:**
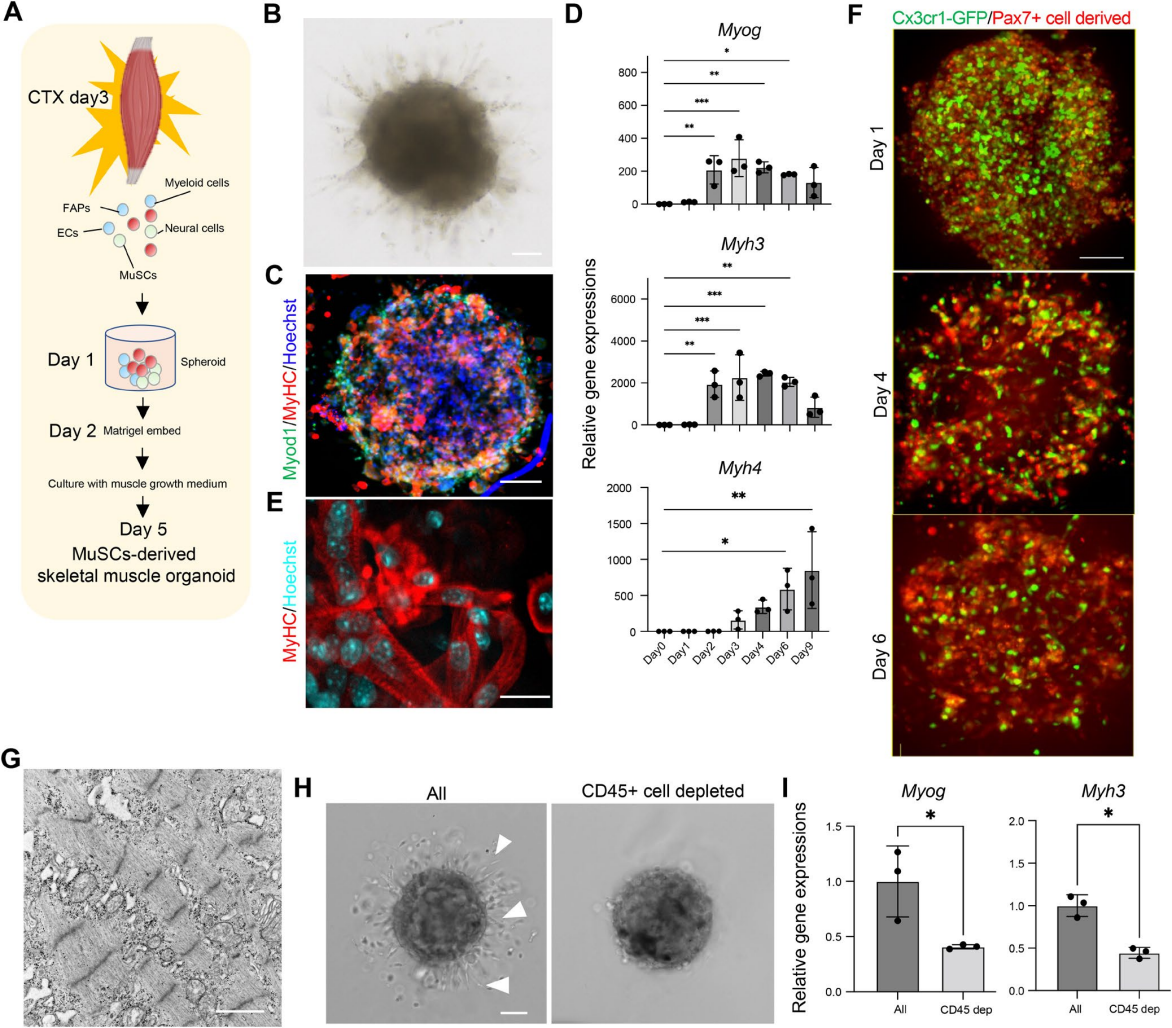
The organoid system recapitulates skeletal muscle regeneration through interaction with macrophages. (A) Schematic overview of establishing the skeletal muscle organoid system. (B) Bright‐field image of a skeletal muscle organoid. Scale 100 μm. (C) Fluorescent staining of MyoD and MyHC in a skeletal muscle organoid. Scale: 100 μm. (D) Gene expression of *Myog*, *Myh3*, and *Myh4* in skeletal muscle organoids during skeletal muscle regeneration (day 0–9). *n* = 3. Data are expressed as the means ± SD. *p < 0.05 vs. Day 0 organoids (one‐way ANOVA with Dunnett's multiple comparisons test). Only significant comparisons are shown. (E) High‐magnification image of MyHC staining in a skeletal muscle organoid. Scale: 20 μm. (F) ^T2‐^CreER‐based PAX7‐tdTomato tracing of skeletal muscle organoids containing CX3CR1‐GFP‐expressing macrophages during skeletal muscle regeneration (days 1–6). Scale 100 μm. (G) SEM image of skeletal muscle organoid. Scale: 1 μm. (H) Bright‐field of skeletal muscle organoids lacking CD45‐expressing cells. Arrowheads indicate differentiated muscle fibers. Scale 100 μm. (I) Gene expression of *Myog* and *Myh3* in control or CD45+ cell‐depleted organoids. *n* = 3. Data are expressed as the means ± SD. *p < 0.05 (unpaired two‐tailed Student's *t*‐test).

To understand the intercellular communication between MuSCs and the Mac_1 macrophage subpopulation that is necessary for muscle regeneration, we established a 3D muscle organoid from mouse primary cells. Three days after CTX injection into the TA muscles in mice, cells from the damaged tissue, including MuSCs, FAPs, immune cells, blood vessel constituent cells, fibroblasts, and nervous system cells, were isolated. Large muscle fibers were excluded using a strainer, and the remaining cells were aggregated and cultured to create mouse muscle organoids (Figure 4A). On day 6 of culture, muscle organoids had formed and exhibited outwardly radiating muscle fibers accompanied by the expression of MyoD and MyHC (Figure 4B,C). The muscle organoids recapitulated the myotube differentiation process in vitro, as evidenced by the sequential mRNA expression of the myogenic regulatory factor *Myog* and the differentiation markers *Myh3* and *Myh4* (Figure 4D). On day 13 of culture, the organoids contained the rhabdomere and sarcomere structures characteristic of mature muscle fibers (Figure 4E,G).

To evaluate the intercellular communication between MuSCs and macrophages in vitro, we used fluorescently labeled skeletal muscle cells to generate muscle organoids. Mice derived from crosses between *PAX7‐Cre* ^*ERT2*^: *LSL‐tdTomato* mice labeled for MuSCs and *CX3CR1‐GFP* mice labeled for macrophages were treated with CTX and tamoxifen, after which single‐cell populations were collected from skeletal muscle 3 days after injury (Figure 4F). Our fluorescence imaging showed abundant tdTomato‐positive MuSCs and GFP‐positive macrophages within the organoids the day after cell aggregation, after which the tdTomato‐positive cells differentiated into myotubes (Figure 4F). These data obtained at the start of culture confirmed that the organoids contained both MuSCs and macrophages. It was also confirmed that skeletal muscle fibers within the organoids obtained at the end of the culture were derived from originally Pax7‐positive MuSCs at the start of the culture and not from residual skeletal muscle fibers present from the beginning.

We then used the organoid model to assess the role of leukocytes during muscle regeneration. Control organoids, which contained all the cell types derived from the injured muscle tissue, exhibited outwardly extending muscle fibers on day 6 of culture. However, muscle organoids prepared from cells depleted of CD45^+^ leukocytes were impaired and had not developed myotubes by day 6 of culture (Figure 4H). Moreover, induction of *Myog* and *Myh3* was significantly diminished in the impaired muscle organoids (Figure 4I). To further examine the role of HGF/MET signaling in muscle regeneration within the organoid model, we treated the organoids with the MET inhibitor PHA‐665752, as used previously in vivo (Figure 3H,I). PHA‐665752 treatment significantly impaired myotube formation on day 4 of culture, as evidenced by a reduction in MyHC‐positive myotubes (Figure S6A,B). In addition, *Myf5* expression on day 1 and *Myog* expression on day 4 were significantly reduced in PHA‐665752‐treated organoids compared to controls (Figure S6C), indicating impaired myogenic progression. These results further support the notion that c‐Met signaling is crucial in muscle regeneration within the organoid model.

### 2.8 Mac_1 Promotes the Proliferation of MuSC in Muscle Organoids

**FIGURE 5:**
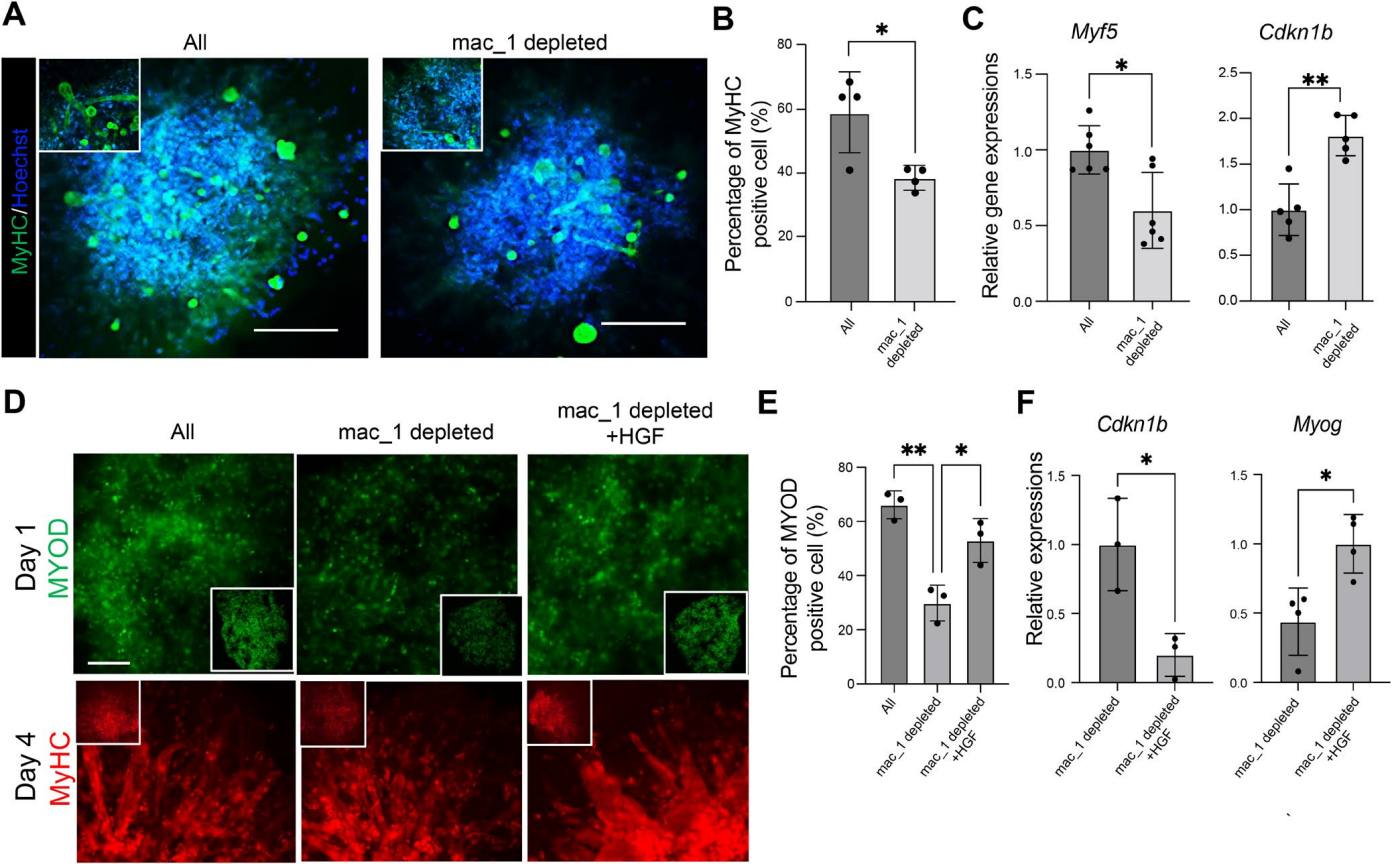
Mac_1 was responsible for promoting skeletal muscle regeneration through *cdkn1b* gene suppression in the organoid system. (A) Staining of MyHC in organoids lacking Mac_1 (F4/80^+^/CD206^dim^/Ly6C^low^/CD9^low^ Cells) on day 4 of culture. Scale 100 μm. (B) Quantification of the ratio of MyHC‐positive cells (cells within green color areas) in (A). *n* = 4. Data are expressed as the means ± SD. *p < 0.05 (unpaired two‐tailed Student's *t*‐test). (C) Gene expression of *Myf5* and *Cdkn1b* evaluated using qPCR. Total RNA was obtained from skeletal muscle organoids depleted of Mac_1 (F4/80^+^/CD206^dim^/Ly6C^low^/CD9^low^ Cells) on day 1 of culture and subjected to analysis. *n* = 6. Data are expressed as the means ± SD. *p < 0.05 (one‐way ANOVA with Tukey's multiple comparisons tests). (D) Staining of MyoD (day 1 after aggregating the cells) and MyHC (day 4 after aggregating the cells) in the skeletal muscle organoids lacking Mac_1, with or without HGF. Scale: 100 μm. (E) Percentage of MyoD‐positive cells in skeletal muscle organoids depleted of Mac_1 with or without HGF in (D). *n* = 3. Data are expressed as the means ± SD. *p < 0.05 (one‐way ANOVA with Tukey's multiple comparisons test). (F) Gene expression of *Cdkn1b* on day 1 and *Myog* on day 4 in skeletal muscle organoids lacking Mac_1, with or without HGF. *n* = 3 for day1 and *n* = 4 for day4. Data are expressed as the means ± SD. *p < 0.05 (unpaired two‐tailed Student's *t*‐test).

To investigate the role of Mac_1 during skeletal muscle regeneration using the organoid model, skeletal muscle organoids were created from all cells collected from injury sites on day 3 post‐injury, as well as from cells in which the Mac_1 fraction was eliminated. Myotube differentiation within these organoids was then compared. Immunofluorescent imaging of day‐4 organoids indicated that the expression of MyHC, a mature myotube marker, was decreased in the organoids lacking the Mac_1 fraction (Figure 5A,B). Myf5, which is widely recognized to be a marker of stem cells activated during the muscle regenerative process, plays a vital role in the early stages of muscle regeneration (Cooper et al. 1999; Ustanina et al. 2007; Wang et al. 2022). In addition, *Cdkn1b*, a negative cell cycle regulator, reportedly plays a central role in the regulatory mechanism by which quiescent stem cells are activated and initiate proliferation (Dumont et al. 2015; Machida et al. 2003). We also used the organoid model to investigate the expression dynamics of those two genes and examined the functional significance of each gene at the early stage of skeletal muscle regeneration. Removal of the Mac_1 fraction reduced the expression of *Myf5* and enhanced the expression of *Cdkn1b* on day 1 of culture, and myotube formation was impaired (Figure 5A–C). This indicates that removing the Mac_1 fraction alone impairs myotube formation, which highlights the importance of Mac_1 to muscle regeneration.

### 2.9 Administration of Exogenous HGF Partially Rescues Muscle Regeneration After Macrophage Depletion

The findings that macrophage (particularly Mac_1)‐derived HGF promotes muscle regeneration by stimulating the proliferation of MuSCs via HGF/MET signaling and that HGF expression decreases with age led us to test whether macrophage‐induced activation of HGF/MET signaling promotes muscle regeneration.

The effect of HGF administration was tested using muscle organoids in which the Mac_1 fraction was excluded. In organoids lacking Mac_1, the numbers of MyoD‐positive cells were reduced, but the reduction was reversed by exogenous administration of HGF (Figure 5D,E). MyHC expression and *Myog* mRNA expression on day 4 were also increased by the administration of HGF (Figure 5D–F). Moreover, exogenous administration of HGF also decreased *Cdkn1b* expression on day 1 of culture and restored the proliferation of MuSCs (Figure 5F). In sum, HGF suppressed *Cdkn1b* expression in MyoD‐positive MuSCs within skeletal muscle organoids and promoted myotube differentiation, as indicated by an increase in MyHC‐positive fibers.

**FIGURE 6:**
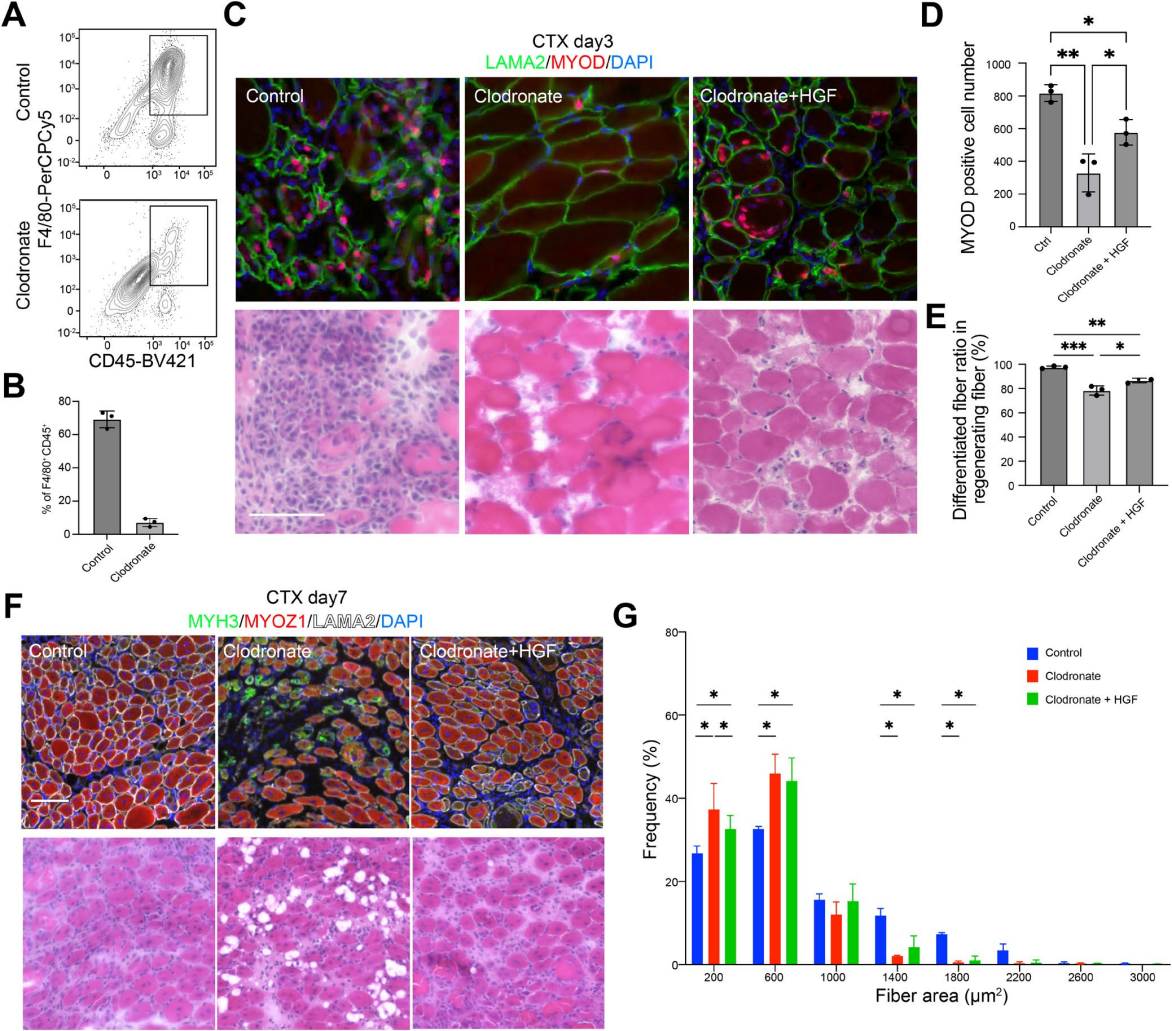
HGF/MET signaling rescues skeletal muscle regeneration due to macrophage dynamic changes in vivo. (A) Flow cytometric analysis shows macrophage depletion after injection of clodronate liposomes. (B) Quantifying percentages of CD45^+^/F4/80^+^ macrophages among live single cells in (A). Data are expressed as the means ± SD. *n* = 3. *p < 0.05 (unpaired two‐tailed Student's *t*‐test). (C) Effect of clodronate administration with or without HGF on MyoD‐ and LAMA2‐positive cells in skeletal muscle on day 3 after CTX administration in vivo. Scale: 100 μm. (D) Effect of clodronate administration with or without HGF on MYOZ1‐positive regenerated and MYH3‐positive regenerating fiber ratios in skeletal muscle tissue sections on day 7 after CTX administration in vivo. Scale: 100 μm. (E) Quantification of MyoD‐positive cell numbers in skeletal muscle tissue after clodronate administration with or without HGF on day 3 after CTX administration in vivo. Cell numbers were counted per unit area of each section. *n* = 3 mice. Data are expressed as the means ± SD. *p < 0.05 (one‐way ANOVA with Tukey's multiple comparisons tests). (F) Quantification of differentiated fiber ratios in skeletal muscle tissue after clodronate administration with or without HGF on day 7 after CTX administration in vivo. *n* = 3. Data are expressed as the means ± SD. *p < 0.05 (one‐way ANOVA with Tukey's multiple comparisons test). Scale: 100 μm. (G) Distribution of muscle fiber cross‐sectional areas. The mean number of muscle fibers within the indicated areas per 2000 myofibers/condition. Data are expressed as the means ± SD. *p < 0.05 (one‐way ANOVA with Tukey's multiple comparisons test).

Previous studies suggested that macrophages are necessary for muscle regeneration after injury (Liu et al. 2017; Oishi et al. 2024). Our findings so far indicate that macrophage proliferation and accumulation become impaired with aging and that HGF secreted by Mac_1 is essential to promote regeneration. We then addressed whether exogenous HGF can reverse the muscle regeneration defect caused by macrophage depletion. To test this, clodronate liposomes were injected into 8‐week‐old mice to deplete the macrophages within regenerating skeletal muscle tissue. Clodronate administration reduced macrophage accumulation (Summan et al. 2006) and the presence of MyoD‐positive cells on day 3 post‐injury (Figure 6A–D). In addition, the percentage per section of myofibers expressing MYOZ1 (Yoshimoto et al. 2020), a marker of mature regenerated myofibers, relative to those expressing MYH3, a marker of immature regenerating myofibers, was reduced, suggesting regeneration was delayed (Figure 6E). Injections of HGF were then made into the TAs at a dose of 0.5 μg/day every other day starting the day after injury, with PBS administered as a control (Convente et al. 2018; Tirone et al. 2019). On day 3 of regeneration, the frequency of MyoD‐positive cells within muscle tissue was partially restored by HGF administration. HGF administration also partially restored the percentage of MYOZ1‐positive mature myofibers in the regenerated day 7 muscle tissue and decreased the ratio of myofibers thinner than 400 μm (Figure 6F,G). Thus, the regeneration defect caused by macrophage depletion was partially reversed by HGF administration.

Following macrophage depletion with clodronate, the delayed muscle regeneration and prolonged inflammation at the site of injury were reminiscent of the conditions observed at the site of injury in older mice (Figure 1J, Figure S2). Given that skeletal muscle regeneration is promoted by HGF derived from macrophages, it was hypothesized that HGF could mitigate the age‐related decline in muscle regeneration. To test that idea, HGF was administered to aged mice, employing a protocol like that used with young mice administered clodronate. Without HGF, aged mice exhibited a smaller percentage of MYOZ1‐positive regenerated mature fibers on day 7 after injury, indicating delayed regeneration. HGF treatment increased the percentage of MYOZ1‐positive fibers and decreased the proportion of thin muscle fibers (< 400 μm^2^), suggesting that HGF can reverse age‐related impairment of muscle regeneration (Figure S6).

## 3 Discussion

This study provides proof of concept for a mechanism by which a specific macrophage subpopulation regulates skeletal muscle regeneration through ligand receptor‐mediated, cell–cell interactions with skeletal muscle stem cells. Moreover, this regulatory mechanism appears to be responsible for the age‐related slowing of skeletal muscle regeneration. Our scRNA‐Seq analysis identified seven macrophage subpopulations within regenerating mouse skeletal muscle and found that these subpopulations change in number, complexity, and gene expression profile with age (Figure 1I–N). In addition, in an in vitro organoid system where cell–cell interactions between macrophages and MuSCs recapitulated skeletal muscle regeneration, the Mac_1 macrophage subpopulation suppressed the expression of the growth inhibitor gene *Cdkn1b* via HGF/c‐MET signaling in MuSCs (Figure 5). However, HGF expression was found to be downregulated in skeletal muscle macrophages from aged mice. Administration of exogenous HGF rescued the diminished skeletal muscle regeneration in macrophage‐depleted and aged mouse models, implicating changes in macrophage‐derived HGF/c‐MET signaling in the slowing of skeletal muscle regeneration associated with aging.

Five of the seven macrophage subpopulations identified in regenerating skeletal muscle did not fit the pro‐inflammatory (Mac_4) or anti‐inflammatory (Mac_5) macrophage phenotype. One of those subpopulations (Mac_0) expresses the senescence‐specific marker GPNMB, which has recently received attention as a target for senolytics and is associated with senescence‐like features (Suda et al. 2021). Although senescent cells are typically associated with detrimental effects on regeneration, they may also have beneficial roles in tissue repair and wound healing (Elder and Emmerson 2020). Consistent with earlier findings (Krasniewski et al. 2022; Moiseeva et al. 2023), Mac_0 macrophages were three times more abundant in homeostatic skeletal muscle from aged than from young mice and were significantly increased in regenerating skeletal muscle, regardless of age. The accumulation of senescent cells within tissues mediates a decline in the function of surrounding cells through the release of fluid‐borne factors such as senescent‐associated secretory protein (SASP). Recent studies have also shown that senescent cells inhibit MuSC proliferation, leading to a delay in skeletal muscle regeneration in both young and aged mice (Moiseeva et al. 2023). Still, the extent of the contribution of senescent macrophages to the slowing of skeletal muscle regeneration and the mechanism by which they act remain unknown. In addition, the Mac_6 subpopulation is 7 times more abundant in aged than in young mice. This subpopulation strongly expresses pro‐inflammatory markers such as *S100a9*, *Itgal*, and *Pglyrp1*. Thus, macrophage subpopulations with both inflammatory and aging phenotypes may contribute to age‐related skeletal muscle dysfunction.

Our study found that ribosome‐related gene expression was reduced in aged macrophages during skeletal muscle regeneration (Figure 1M). Given that ribosome biogenesis is essential for cellular growth and proliferation, this reduction may reflect an impaired ability of aged macrophages to support the regenerative process. Consistent with earlier findings (Krasniewski et al. 2022; Wang et al. 2020), the present study identified macrophages (Mac_3) that actively proliferate within skeletal muscle. We also observed that the Mac_3 population increases significantly during regeneration in skeletal muscle and that it decreases with age (Figure 1K). These findings suggest that the age‐related decline in ribosome biogenesis may lead to reduced proliferation of macrophage subpopulations, such as Mac_3, ultimately contributing to the impaired skeletal muscle regeneration observed in aged mice.

Macrophage diversity has been investigated in mice with severe muscle damage from lacerations and in MDX mice, a model of muscular dystrophy, which disrupts skeletal muscle regeneration by promoting chronic inflammatory cell infiltration, excessive fibrosis, abnormal myogenesis, and vascular disorganization (Mojumdar et al. 2014; Novak et al. 2014). Mac_1 macrophages are closely related to the hybrid type in that study and express markers found in all macrophage subpopulations, including the M1 and M2 markers. Unlike in earlier studies, we followed Mac_1 during the period of skeletal muscle regeneration when proliferation was active and observed it positively affect regeneration by enhancing MuSC proliferation. Aging altered the function of Mac_1 (Figure 1N), suggesting the function of hybrid macrophages may also change with aging and under disease conditions from having a positive effect on skeletal muscle regeneration to a negative effect. Future analysis of hybrid macrophages at different times and in different models will provide a deeper understanding of macrophage function.

Previous studies have shown that HGF facilitates skeletal muscle regeneration (Sisson et al. 2009) and that c‐Met, the HGF receptor, is essential for the activation of MuSCs (Webster and Fan 2013). More recently, it was shown that HGF also induces polarization of macrophages to the M2 phenotype during skeletal muscle regeneration (Choi et al. 2019). However, it was unclear whether and how HGF secreted by macrophages directly regulates MuSCs and promotes skeletal muscle regeneration. Using an in vivo mouse model and skeletal muscle organoids in the present study, we obtained evidence that macrophages (Mac_1 in particular) secrete HGF and promote MuSC proliferation by suppressing *Cdkn1b* expression, thereby inducing skeletal muscle regeneration (Figure 5). Expression of macrophage‐derived HGF was reduced in the skeletal muscle of aged mice, and exogenous HGF administration to aged mice partially reversed the aging‐related reduction in skeletal muscle regeneration. This suggests that changes in macrophage‐derived HGF expression are involved in the onset and progression of age‐related impairment of skeletal muscle regeneration. Recently, HGF was identified as a plasma protein responsible for sarcopenia in a genome‐wide association study in sarcopenia patients (Jiang et al. 2023). Plasma HGF levels are lower in patients with sarcopenia than in healthy controls, and the results of our study suggest that this reduction may be due to decreased synthesis and secretion by macrophages. This makes HGF and HGF‐secreting macrophages potential therapeutic targets for treating sarcopenia in humans. Other studies have also reported that age‐related nitration of tyrosine residues in extracellular HGF results in the loss of c‐met binding capacity and muscle tissue homeostasis (Elgaabari et al. 2024). It is thus becoming clear that HGF is a critical factor in age‐related skeletal muscle homeostasis and regeneration and a potential therapeutic target for the treatment of sarcopenia. As a natural bioactive protein, the use of HGF itself has drawbacks in terms of production cost and the need to combine it with drug delivery technology. However, there are reports of the successful creation of artificial MET agonists (artificial HGF) to replace HGF (Ito et al. 2015), and it is anticipated that a small‐molecule MET agonist useful for the treatment and prevention of sarcopenia will be developed.

In the present study, we developed skeletal muscle organoids that enabled us to evaluate the function of macrophage subpopulations during skeletal muscle regeneration. While model systems that reproduce skeletal muscle regeneration have been reported previously, most require unique culture methods and techniques and focus on the contraction and strength of skeletal muscle, rarely focusing on cell–cell interaction among multiple cells during skeletal muscle regeneration (Cho and Jang 2021; Kase et al. 2023). By contrast, our innovative muscle organoid model is a simple culture system that requires no special equipment and enables recapitulation of the cell–cell interactions among multiple cell types. The initial step involves passing cells through strainers to eliminate damaged and dead myofibers. Consequently, this model system does not include phagocytosis of damaged myofibers by macrophages; however, the activation, proliferation, and differentiation of MuSCs can be visualized (Figure 4). Within the organoid system, MuSCs and macrophages coexist most abundantly on the day after the cells are collected and aggregate during the proliferative phase before differentiation proceeds (Figure 4). This indicates that cell–cell interactions play crucial roles within organoids during the proliferative phase of skeletal muscle regeneration. Consequently, this model provides an excellent opportunity to investigate the effects of interaction between MuSCs and macrophages on skeletal muscle regeneration through MuSC proliferation. This model is also suitable for a wide range of skeletal muscle studies, as it enables functional evaluation of multiple cell interactions, particularly during the proliferative phase, without the need for complex manipulations.

## 4 Limitations of This Study

Mac_1 promoted muscle regeneration and repair by modulating MuSC proliferation in a skeletal muscle organoid model. It should be noted, however, that this model does not fully recapitulate skeletal muscle regeneration in vivo. While the effects on proliferation are strongly reflected, the effects on differentiation are limited. Although FAPs promote skeletal muscle regeneration by producing extracellular matrix, the use of Matrigel, an extracellular matrix substrate, in this model may have reduced the impact of FAPs on skeletal muscle regeneration. Regarding vascular endothelial cells, the absence of in vivo vascular functionality, such as nutrient supply from the blood, in the organoids may have contributed to the present findings. More precise model systems will be required to study the mechanisms of skeletal muscle regeneration through cooperation between macrophages, MuSCs, and other cell types. In addition, it should be noted that HGF expression was also observed in the Mac_0 subpopulation, suggesting that the removal of Mac_1 in this study did not completely suppress HGF/MET signaling. This study was carried out primarily in mice, so the role of macrophage subpopulations in human skeletal muscle regeneration remains to be determined. RNA‐Seq analysis targeting human skeletal muscle macrophages and experiments using human cells are expected to clarify the effects of macrophage subpopulations on human skeletal muscle regeneration.

One limitation of this study is that we primarily relied on the CTX‐induced regeneration model to investigate macrophage subpopulations and their role in skeletal muscle regeneration. While the CTX model is widely used to study muscle regeneration due to its reproducibility and well‐characterized regenerative timeline (Wang et al. 2022), it primarily induces acute muscle injury and may not fully recapitulate the chronic regenerative deficits observed in other models, such as BaCl_2_‐induced injury, denervation, or age‐related muscle degeneration (Cui et al. 2023; Hardy et al. 2016). Therefore, further studies are needed to determine whether the macrophage subpopulations and mechanisms identified in this study play similar roles in other models of muscle regeneration, including those associated with aging‐related decline.

Despite the limitations of this study, it has been demonstrated that heterogeneous macrophage subpopulations are present in skeletal muscle during the regenerative process and that the dynamics of those subpopulations change significantly with age. Some subpopulations also regulate MuSC status through ligand–receptor signaling to promote skeletal muscle regeneration, ameliorating age‐related defects. In summary, it is anticipated that targeting specific macrophage subpopulations and their signaling pathways will contribute to the development of effective strategies for preventing and treating sarcopenia.

## 5 Materials and Methods

All experimental procedures were conducted according to a protocol approved by the President of Nippon Medical School after being reviewed by the Nippon Medical School Animal Care and Use Committee (Approval No. 30–027) and adhered to the relevant guidelines and regulations concerning the management and handling of experimental animals. This study is reported in accordance with the ARRIVE guidelines (https://arriveguidelines.org).

### 5.1 Animals

C57BL/6J male mice were purchased from Sankyo Labo Service (Japan). Both the young (8‐week‐old) and aged (24‐month‐old) mice used in these experiments were housed at 22°C under a 12‐h light/dark cycle and fed ad libitum.

### 5.2 Muscle Regeneration

To induce muscle injury, mice were anesthetized with isoflurane (1.5%–2%) (Pfizer, NY, USA) and 100 μL of 10 μM cardiotoxin (Sigma‐Aldrich, St. Louis, MO, USA) (CTX) was injected into the tibialis anterior (TA) muscles using a 29 G syringe (Terumo, Tokyo, Japan). Both TA muscles in each mouse were injured and then collected 7 days after CTX injection.

### 5.3 Histological Staining

For hematoxylin/eosin staining, TA muscles were fixed by immersion in Tissue‐Tek Ufix (Sakura Finetek, Tokyo, Japan), embedded in paraffin, and cut into 10‐μm‐thick sections. The sections were then deparaffinized, rehydrated, stained with hematoxylin for 3 min, washed with running water for 15 min, stained in eosin for 15 min, and quickly washed in an ethanol series (70%, 80%, 90%, 95%, and 100%) before finally washing twice in xylene. Images of the hematoxylin/eosin‐stained sections were then acquired using a microscope (BZ‐X810, Keyence, Osaka, Japan) and analyzed to assess the minor fiber axis and to quantify regenerating and necrotic fibers.

For immunostaining, TA muscles were isolated 0, 1, 3, 5, or 7 days after CTX injection, frozen in cooled isopentane in liquid nitrogen, cut into 10 μm‐thick cryosections, and stained for the antibodies listed in Table S2 and with Hoechst 33342 (Thermo Fisher Scientific). Images of stained sections were acquired using a confocal microscope (Oxford Instruments Andor, Northern Ireland) or BZ‐X810 microscope.

### 5.4 Injection of PHA‐66752

PHA‐665752 (Selleck Chemicals, USA), a c‐met inhibitor, was dissolved in 2% DMSO (Sigma Aldrich, USA) in corn oil (FUJIFILM Wako Pure Chemical Corporation, Japan) and intraperitoneally administered to mice daily at a dose of 20 mg/kg. Administration was performed from the day of CTX‐induced skeletal muscle injury to day 3.

### 5.5 In Vivo BrdU Labeling

Proliferating cells were detected using a FITC BrdU Flow Kit (BD Biosciences, USA). The cells were labeled in vivo through intraperitoneal injection of bromodeoxyuridine (BrdU) in PBS at a dose of 50 mg/kg on day 3 after CTX injection. 1 h after the BrdU injection, TA tissues were isolated, fixed, permeabilized, and stained with FITC‐conjugated anti‐BrdU antibodies according to the instructions in the FITC BrdU Flow Kit.

### 5.6 Injection of Clodronate‐Encapsulated Liposomes

Clodronate liposomes were used to generate a macrophage depletion model described previously (Liu et al. 2019; Summan et al. 2006). Clodronate liposomes and control liposomes were purchased from Katayama Chemicals (Japan). Clodronate liposomes or control liposomes (40 μL) were diluted to 100 μL with PBS and injected intraperitoneally 48 h before CTX injection and on days 0 and 3 after CTX administration.

### 5.7 Injection of HGF

One group of mice received 20 μg/kg injections of HGF (R & D, Minneapolis, MN) in 50 μL of saline daily into both TA muscles on day 1 after CTX injection. For all experimental injections, the same volume of saline was injected into the TA muscles of the mice that served as controls.

### 5.8 Sample Processing for Flow Cytometry

Samples were prepared for fluorescence‐activated cell sorting (FACS), as described previously (Watanabe et al. 2023). Isolated TA muscles from each mouse were homogenized and digested with collagenase II solution (2 mg/mL, Worthington Biochemical, Lakewood, NJ, USA) for 1 h at 37 C in a shaker. The sample was then neutralized with FACS buffer (PBS with 2% FBS and 1X pen/strep) and centrifuged at 500× g for 3 min, and the supernatant was removed. The cell suspensions were passed through 100‐μm strainers, centrifuged, aspirated, resuspended, and passed through 40‐μm strainers. Pelleted cells were resuspended in PBS with 2% FBS for antibody staining and analysis. The following antibodies directed against mouse antigens were used: BV421‐conjugated CD45 (clone 30‐F11, BioLegend, San Diego, CA, USA), PECy7‐conjugated CD11b (clone M1/70, BD Biosciences, Franklin Lakes, NJ, USA), APCCy7‐conjugated Ly6G (clone 1A8, BioLegend), APC‐conjugated Ly6C (clone HK1.4, BioLegend), PE‐conjugated F4/80 (clone T45‐2342, BD Biosciences), FITC‐conjugated Siglec‐F (clone S17007L, BioLegend), and PE‐conjugated CXCR2 (clone SA044G4, BioLegend). Data were acquired with a FACSAria III (BD, Franklin Lakes, NJ, USA) and LSRFortessa (BD) and analyzed using FlowJo v10 (Treestar, San Francisco, CA, USA). In addition, the numbers of live cells were counted. The absolute value of each cell count was calculated by multiplying the percentage of the total cells counted that were alive by the number of live cells. Cell counts for muscle tissue are expressed per unit tissue weight, peripheral blood cell counts are expressed per unit blood volume, and bone marrow cell counts per tibia.

### 5.9 Single‐Cell RNAseq

Live (7AAD‐negative) leukocytes (CD45‐positive), fibro‐adipogenic progenitors (Ly6A/E‐positive), endothelial cells (CD31‐positive), and skeletal muscle stem cells (CD106‐positive) were sorted from mouse TA muscle tissue with FACS on days 0 and 3 of regeneration. Cells were then processed for droplet‐based scRNA‐seq. Single‐cell capturing and library generation were performed with Chromium Single Cell 3’ Reagents Kits v3 (10× Genomics, CA, USA) using the manufacturer's protocol. The sorted cells were resuspended at a concentration of approximately 1000 cells/μL in PBS containing 0.1% BSA, and we targeted 10,000 cells per sample for capture.

A single cell suspension was mixed with RT‐PCR master mix and loaded with Single Cell 3′ v3 Gel Beads and Partitioning Oil onto a Chromium Chip B. The chip was then loaded onto a Chromium Controller for single‐cell GEM generation. Immediately following GEM generation, the Gel Bead is dissolved, primers are released, and any co‐partitioned cell was lysed. Incubation of the GEMs produces barcoded, full‐length cDNA from poly‐adenylated mRNA.

After GEM disruption, full‐length cDNA was amplified to generate a library. Finally, amplified cDNAs were fragmented, and adapter and sample indexes were added to the finished libraries. The size profiles of the pre‐amplified cDNA and sequencing libraries were examined using an Agilent Bioanalyzer 2100 with a High Sensitivity DNA chip (Agilent Technologies, CA, USA). Libraries were sequenced on an Illumina Hiseq 1500 (Illumina, CA, USA) and HiSeq X Ten (Illumina, CA, USA).

### 5.10 Processing of scRNA‐Seq Data

Cell Ranger software was used for demultiplexing, barcode processing, gene counting, and aggregation (v3.0.2 for young mouse samples and v3.1.0 for aged mouse samples). Cell Ranger mkfastq was used to convert the barcode and read data to FASTQ files. Cell Ranger count was used to identify cell barcodes aligned to an indexed mm10 genome.

### 5.11 Dimensionality Reduction and Clustering

We performed unsupervised clustering and differential gene expression analyses in the Seurat (Hao et al. 2021) package (4.1.1). The data from all samples were combined to generate an aggregate Seurat object. The object was created by filtering cells with > 500 detected genes and a mitochondrial gene percentage < 7. After principal component analysis was performed and a resolution = 0.2 was set, we obtained clusters for each sample. These clusters were also identified based on the marker genes of the major cell types. Unbiased clustered macrophage/DC and myogenic cell populations were subsetted for further subclustering analysis.

### 5.12 Cell Communication Network Analysis

CellPhone DB v2.0 is a publicly available repository of curated receptors, ligands, and interactions (Efremova et al. 2020; Vento‐Tormo et al. 2018). Although it was created using human‐specific ligand–receptor interactions, it can be applied to mouse datasets by mapping human genes to their biomaRT (2.52.0). We used CellPhoneDB for the analysis of cellular crosstalk between macrophage/DC subclusters and myogenic cell subclusters, and interactions in which the macrophage/DC side became a ligand were extracted. Significant cell–cell interactions were selected with a *p*‐value < 0.001.

### 5.13 Quantitative RT‐PCR

Total RNA was isolated from homogenized TA muscles using ISOGEN (Nippon Gene, Tokyo, Japan). Following the manufacturer's instructions, the RNA was isolated using the phenol‐chloroform extraction and isopropanol precipitation protocol. Total RNA was extracted from sorted cells using a NucleoSpin RNA Plus XS (Takara, Japan). Complementary DNA (cDNA) was synthesized using SuperScript IV VILO Master Mix with ezDNase (Thermo Fisher Scientific, USA). cDNA was analyzed using real‐time PCR in a QuantStudio 5 Real‐time PCR system (Applied Biosystems, Foster City, CA, USA) with PowerTrack SYBR Green Master Mix (Applied Biosystems). The primers used for qPCR are listed in Table S1. Glyceraldehyde‐3‐phosphate dehydrogenase (Gapdh) expression was used as an internal control.

### 5.14 Organoid Culture

Isolated TA muscles were homogenized on CTX day 3 and digested with collagenase II solution (2 mg/mL, Worthington Biochemical, Lakewood, NJ, USA) for 1 h at 37°C in a shaker. The cell suspensions were passed through 100‐μm strainers, centrifuged, aspirated, resuspended, and passed through 40‐μm strainers. Pelleted cells were then resuspended in PBS with 2% FBS for antibody staining for flow cytometric sorting.

After resuspending cells in Advanced DMEM/F12 containing 50% FBS and 10 μM Y27632, 2×10^5^ cells/well were seeded into v‐bottom low‐attachment wells in a 96‐well plate. The plate was then spun down in a centrifuge, and the culture was placed in a CO_2_ incubator overnight. Generated muscle spheroids were transferred to wells with a solidified Matrigel bed, after which medium was added to the wells, and the spheroids were cultured for 7 days.

### 5.15 Transmission Electron Microscopy (TEM)

Samples for TEM were fixed in phosphate‐buffered 2% glutaraldehyde (Electron Microscopy Science, PA) and post‐fixed in 2% osmium tetroxide (Heraeus Chemicals South Africa, South Africa) for 2 h in an ice bath. The specimens were dehydrated in a graded ethanol series (FUJIFILM Wako Pure Chemicals Corporation, Osaka, Japan) and embedded in epoxy resin (TAAB laboratories, UK). Ultrathin sections were cut using an ultramicrotome, stained with uranyl acetate for 15 min and lead staining solution for 5 min, and examined using TEM (HITACHI H‐7600).

### 5.16 Satellite Cell Isolation and Culture Conditions

Mouse primary SCs were isolated from the TA muscles of male mice. After excess fat, connective tissue, and tendons were removed, hindlimb muscles were minced and digested in 0.2% collagenase type II (Worthington Biochemical Corp., Freehold, NJ) for 1 h at 37°C. Mononuclear cells were stained with PE‐Cy7‐conjugated anti‐CD31, anti‐CD45, and anti‐Ly6A/E as well as with FITC‐conjugated antiCD106 antibodies for 30 min on ice and resuspended in PBS containing 2% FBS. SCs were isolated using a FACS Aria III flow cytometer (BD Biosciences). Forward scatter, side scatter, and 7‐AAD gating excluded debris and dead cells. Data were collected using FACS Diva software and FlowJo (BD Biosciences). SCs were cultured in GlutaMax DMEM (Life Technologies, Grand Island, NY) supplemented with 20% FBS, 10 ng/mL basic fibroblast growth factor (Cell Signaling Technology, Beverly, MA), 0.2 μg/cm2 iMatrix‐511 silk (Takara bio), and 1% penicillin–streptomycin at 37°C under 5% CO_2_. Myogenic differentiation was induced in GlutaMax DMEM supplemented with 5% horse serum and 1% penicillin–streptomycin on a Matrigel‐coated plate at 37°C under 5% CO_2_.

### 5.17 Statistics and Reproducibility

Statistical analyses were performed using Graph Pad Prism 10 software. The images were prepared using Adobe Illustrator CS5. Sample sizes were not based on power calculations. Data are presented as the mean ± s.d., except where otherwise indicated. For experiments involving two factors, data were analyzed using two‐way ANOVA followed by Tukey's or Dunnett's post hoc tests, except where otherwise indicated. Individual pairwise comparisons were made using Student's *t*‐test, except where otherwise indicated. Values of *p* < 0.05 were considered significant.

## Author Contributions

H.K., M.S., and R.O. carried out the cell culture. H.K., M.S., R.O., and Y.Y. conducted the histological analysis. H.K. carried out animal experiments, analyzed data, and performed single‐cell RNA sequencing experiments. I.M. and Y.O. analyzed next‐generation sequencing data. H.K. and Y.O. conceived the original idea. H.K., R.O., and Y.O. wrote the manuscript.

## Conflicts of Interest

The authors declare no conflicts of interest.

## Supporting information


Appendix S1.

## Data Availability

All scRNA‐seq data are available in the GEO under accession number GSE272412.
