# Supplementary material for: Macrophage Subpopulation Promotes Skeletal Muscle Regeneration Through HGF/MET Signaling‐Mediated Skeletal Muscle Stem Cell Proliferation
Source: Aging Cell. 2025 Mar 25;24(6):e70042. doi: 10.1111/acel.70042 (PMC12151914; doi:10.1111/acel.70042)
Supplement: Supplementary file 1 — Appendix S1. [file ACEL-24-e70042-s001.docx]

**Supplemental Information**

**Methods**

**Masson-trichrome**

To visualize collagen fibers, tissue samples were stained with Masson Trichrome. Tissue sections were deparaffinized, rinsed under running water, and soaked in mordant (Muto Pure Chemical, Japan). After rinsing under running water, the sections were immersed in Weigert's iron hematoxylin (Muto Pure Chemical, Japan), rinsed with water, then immersed in 0.5% hydrochloric acid alcohol. The sections were again rinsed in running water and soaked in mordant prepared by mixing equal parts of 2.5% phosphomolybdic acid solution and 2.5% phosphotungstic acid solution. The sections were then soaked in orange G solution, washed in 1% acetic acid water, soaked in Masson’s stain A solution, washed in 1% acetic acid, soaked in 2.5% phosphotungstic acid solution, rewashed in 1% acetic acid, soaked in aniline blue, and rewashed in 1% acetic acid. After dehydration, the sections were mounted under coverslips.

**Organoid culture with PHA-66752**

PHA-665752 (Selleck Chemicals, USA), a c-met inhibitor, was dissolved in 2% DMSO (Sigma Aldrich, USA) and added to organoid culture medium with final concentration of 10 μM. The PHA-66752 treatment was performed from the day 0 to day 4 of culture period.

**Supplementary materials**

**Supplementary tables**

**Table S1, Primer sequences for qRT-PCR**

| **Genes** | **Forward** | **Reverse** |
| --- | --- | --- |
| *Met* | GGGAACTGGCTACTGCTCTG | TTCTGCTACACCGTCAGCTTT |
| *Hgf* | TTTGGCCATGAATTTGACCT | AGTGATGGATACCGTCCCTTT |
| *Myog* | CCTTGCTCAGCTCCCTCA | TGGGAGTTGCATTCACTGG |
| *Myh3* | AAAAGGCCATCACTGACGC | CAGCTCTCTGATCCGTGTCTC |
| *Myh4* | GCTTGAAAACGAGGTGGAAA | CCTCCTCAGCCTGTCTCTTG |
| *Myf5* | TGAAGGATGGACATGACGGACG | TTGTGTGCTCCGAAGGCTGCTA |
| *Cdkn1b* | TCAAACGTGAGAGTGTCTAACG | CCGGGCCGAAGAGATTTCTG |
| *Gapdh* | AATGTGTCCGTCGTGGATCT | CATCGAAGGTGGAAGAGTGG |

**Table S2. Reagents and resources for this study**

| REAGENT or RESOURCE | SOURCE | IDENTIFIER |
| --- | --- | --- |
| Antibodies | | |
| Rabbit monoclonal anti-JunB (clone C37F9) | Cell Signaling Technology | Cat#3753; RRID: AB_2130002 |
| Brilliant Violet 510 rat monoclonal anti-mouse/human CD11b (clone M1/70) | BioLegend | Cat# 101245, RRID: AB_2561390 |
| Rabbit polyclonal anti-SEPP1 | Thermo Fisher Scientific | Cat# PA5-112707, RRID: AB_2867442 |
| eFluor 660 rat monoclonal anti-GPNMB (clone CTSREVL) | Thermo Fisher Scientific | Cat# 50-5708-82, RRID: AB_2574239 |
| Rabbit recombinant monoclonal anti-Ki67 (clone SP6) | Abcam | Cat# ab16667, RRID: AB_302459 |
| Rabbit anti-MYOZ1 | Sigma-Aldrich | Cat# HPA038437, RRID: AB_2676009 |
| Rat monoclonal anti-Laminin alpha-2 (clone 4h8-2) | Santa Cruz Biotechnology | Cat# sc-59854, RRID: AB_784266 |
| Rabbit monoclonal anti-Myod1 | Abcam |  |
| Rabbit recombinant monoclonal anti-CCR2 (clone EPR20844-15) | Abcam | Cat# ab273050, RRID: AB_2893307 |
| Rat monoclonal anti-mouse CD206 (clone MR5D3) | Bio-Rad | Cat# MCA2235GA, RRID: AB_322613 |
| Mouse monoclonal anti-myosin, sarcomere | DSHB | Cat# MF 20, RRID: AB_2147781 |
| Mouse monoclonal anti-laminin alpha-1 (clone G-12) | Santa Cruz Biotechnology | Cat# sc-74418, RRID: AB_2133743 |
| Alexa Fluor 647 mouse monoclonal anti-MYH3 (clone F1.652) | Santa Cruz Biotechnology | Cat# sc-53091, RRID: AB_670121 |
| Goat anti-Hepatocyte Growth Factor | R and D Systems | Cat# AF-294-NA, RRID: AB_354451 |
| Goat polyclonal anti-mouse Hgf | R and D Systems | Cat# AF2207, RRID: AB_2118605 |
| Alexa Fluor 488 goat anti-Rat IgG (H+L) | Thermo Fisher Scientific | Cat# A-11006, RRID: AB_2534074 |
| Alexa Fluor 546 F(ab')2-goat anti-Rabbit IgG (H+L) | Thermo Fisher Scientific | Cat# A-11071, RRID: AB_2534115 |
| Alexa Fluor 647-AffiniPure Goat Anti-Rat IgG (H+L) | Jackson ImmunoResearch Labs | Cat# 112-605-167, RRID: AB_2338404 |
| Alexa Fluor 488 goat polyclonal anti-Rat IgG (H+L) | Thermo Fisher Scientific | Cat# A-11006, RRID: AB_2534074 |
| Alexa Fluor Plus 647 donkey polyclonal anti-Mouse IgG (H+L) | Thermo Fisher Scientific | Cat# A32787TR, RRID: AB_2866494 |
| Rat monoclonal anti-EMR1 | Santa Cruz Biotechnology | Cat# sc-52664, RRID: AB_629466 |
| PE rat monoclonal anti-mouse CD31 (clone 390) | BD Biosciences | Cat# 102407, RRID: AB_312902 |
| PE/Cy7 rat monoclonal anti-mouse CD45 (clone 30-F11) | BD Biosciences | Cat# 552848, RRID: AB_394489 |
| APC/Cyanine7 rat monoclonal anti-mouse Ly-6A/E (Sca-1) (clone D7) | BioLegend | Cat# 108125, RRID: AB_10639725 |
| FITC rat monoclonal anti-mouse CD106 (clone 429 (MVCAM.A)) | BioLegend | Cat# 105705, RRID: AB_313206 |
| Fluorescein rat monoclonal anti-mouse Integrin alpha 7 (clone 334908) | R and D Systems | Cat# FAB3518F, RRID:AB_2128442 |
| Alexa Fluor 647 rat monoclonal anti-mouse CD106 (clone 429 (MVCAM.A)) | BioLegend | Cat# 105711, RRID: AB_493430 |
| FITC rat monoclonal anti-mouse Ly-6C (clone HK1.4) | BioLegend | Cat# 128005, RRID: AB_1186134 |
| APC/Cyanine7 rat monoclonal anti-mouse Ly-6G (clone 1A8) | BioLegend | Cat# 127623, RRID: AB_10645331 |
| PE rat monoclonal anti-mouse F4/80 (clone T45-2342) | BD Biosciences | Cat# 565410, RRID: AB_2687527 |
| Brilliant Violet 421 rat monoclonal anti-mouse CD192 (CCR2) (clone SA203G11) | BioLegend | Cat# 150605, RRID: AB_2571913 |
| Brilliant Violet 421 rat monoclonal anti-mouse CD45 (clone 30-F11) | BioLegend | Cat# 103133, RRID: AB_10899570 |
| PE/Dazzle 594 rat monoclonal anti-mouse CD45 (clone 30-F11) | BioLegend | Cat# 103145, RRID: AB_2564002 |
| PE rabbit polyclonal anti-RHAMM/CD168 | Novus | Cat# NBP1-76538PE, RRID: AB_2927540 |
| PE/Dazzle 594 rat monoclonal anti-mouse CD9 (clone MZ3) | BioLegend | Cat# 124821, RRI D: AB_2800601 |
| Alexa Fluor 647 rat anti-mouse CD34 | BD Biosciences | Cat# 560230, RRI D: AB_1645200 |
| Alexa Fluor 647 rat monoclonal anti-mouse CD206 (Mannose Receptor) (clone MR5D3) | BD Biosciences | Cat# 565250, RR ID: AB_2739133 |
| Chemicals, peptides, and recombinant proteins | | |
| Murine FGF-basic | Pepro tech | Cat# 450-33; Accession: P15655 |
| Y-27632 dihydrochloride | Tocris | Cat# 1254; CAS:129830-38-2 |
| RPMI 1640 | Nacalai tesque | Cat# 30264-56 |
| DMEM + GlutaMAX | Gibco | Cat# 10569-010 |
| Penicillin-Streptomycin | Gibco | Cat# 15140122 |
| HEPES solution | Sigma | Cat# H0887-100ML; CAS:7365-45-9 |
| Advanced DMEM/F-12 | Gibco | Cat# 12634-010 |
| GlutaMAX Supplement | Gibco | Cat# 35050061 |
| Collagenase Type2 | Worthington Biochemical Corporation | Cat# LS004177 |
| HBSS(+) | Nacalai tesque | Cat# 09735-75 |
| Matrigel Basement Membrane Matrix | Corning | Cat# 356237 |
| PHA-665752 | Selleckchem | Cat# S1070; CAS: 477575-56-7 |
| Clodronate liposome | Katayama chemical | Cat# 16001003 |
| Control liposome | Katayama chemical | Cat# 16003631 |
| Cardiotoxin | Latoxan | Cat# L8102; CAS: 56574-47-1 |
| Mouse HGF | R&D Systems | Cat# 2207-HG-025; Accession: Q53WS5 |
| Critical commercial assays | | |
| BD Pharmingen™ FITC BrdU Flow Kit | BD Bioscience | Cat# 559619; RRID: AB_2617060 |
| 10x Genomics Chromium Single Cell 3’ | 10X Genomics | PN-1000075 |
| Deposited data | | |
| Single Cell RNA sequence data | This study | GSE272412 |
| Experimental models: Cell lines | | |
| RAW 264.7 | ATCC | Cat# TIB-71 |
| Oligonucleotides | | |
| Gene expression analysis | This study | Table S1 |
| Software and algorithms | | |
| FlowJo | FlowJo | [https://www.flowjo.com](https://www.flowjo.com/) |
| R 4.2.1 | The R Foundation | http://www.r-project.org/ |
| Seurat R package | Hao Y et al. ^50^ | http://satijalab.org/seurat/ |
| GraphPad Prism 10.2.3 | GraphPad Software | https://www.graphpad.com |
| Cell Ranger | 10x Genomics | [https://www.10xgenomics.com](https://www.10xgenomics.com/) |
| ImageJ | ImageJ software | <https://imagej.nih.gov/ij/> |
| Analysis Applications Hybrid Cell Count | Keyence Corporation | https://www.keyence.com |

**Supplementally figures and figure legends**

**
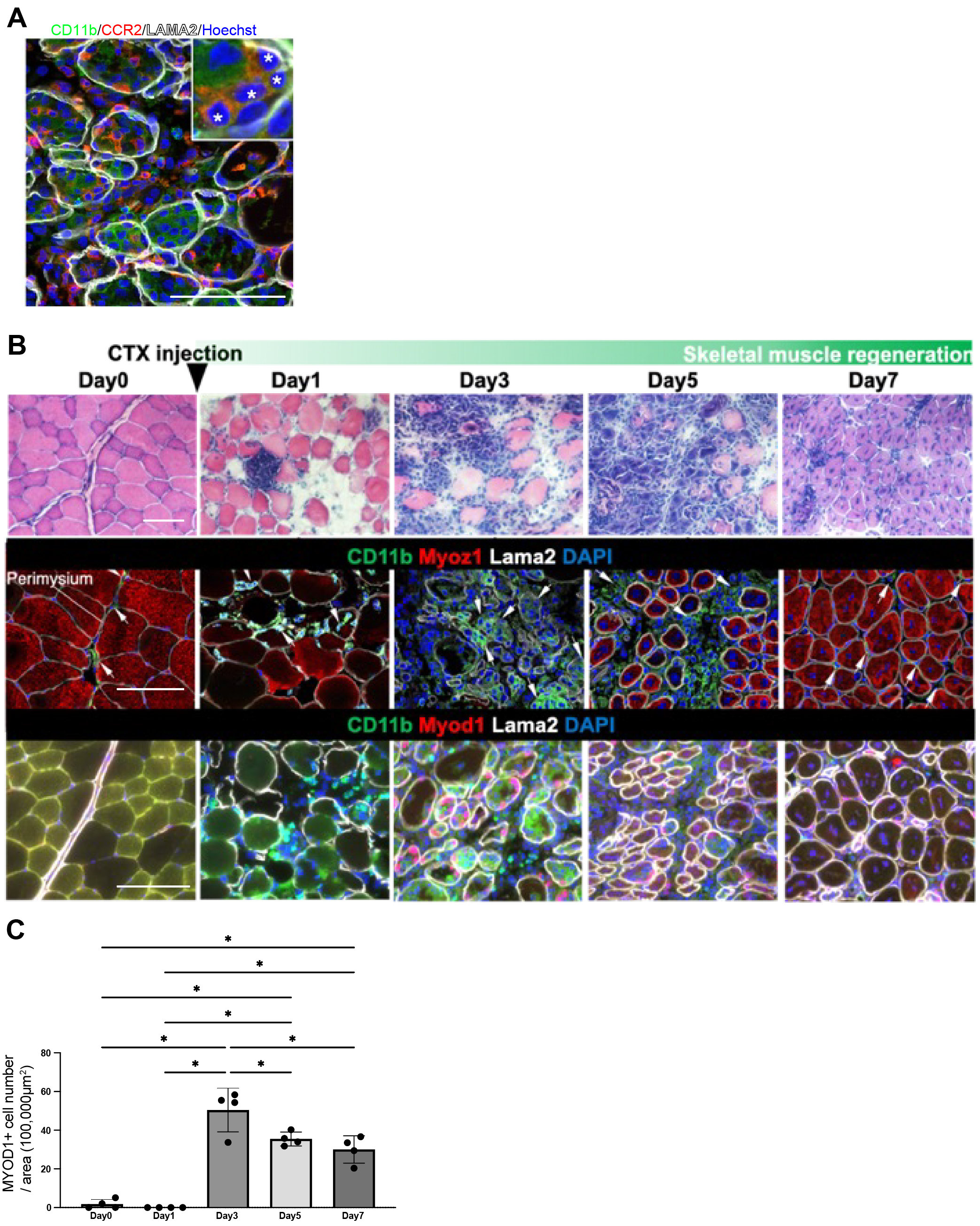
**

**Figure S1, related to Figure 1. Aging delays skeletal muscle regeneration and repair after injury.**

1. Representative image of immunofluorescent staining for CCR2, CD11b and Lama2 in skeletal muscle tissue on day 3 after CTX administration. Scale: 100 μm.
2. Representative image of immunofluorescent staining of CD11b, MYOZ1, LAMA2 and MyoD during skeletal muscle regeneration. Scale: 100 μm
3. MyoD-positive cell number count per unit area (100,000μm^2^) of (B). Data are expressed as the means ± SD. * *p* < 0.05 (one-way ANOVA with Tukey’s multiple comparisons test).

**Figure S2, related to Figure 1. Skeletal muscle regeneration was delayed in aged mice.**

1. H&E staining of young (top) and aged (bottom) TA sections on day 7 after CTX injection. Scales: 100 μm.
2. Masson’s trichrome staining of young (top) and aged (bottom) TA sections on day 7 after CTX injection. Scales: 100 μm.
3. Immunostaining for F4/80 of young (top) and aged (bottom) TA sections on day 7 after CTX injection. Scales: 100 μm.

**Figure S3, related to Figure 2. Mac_1 subpopulation interacted more strongly with myogenic cell populations.**

Dot plot representing a list of ligand receptors between Mac_1 and myogenic subtypes.

**
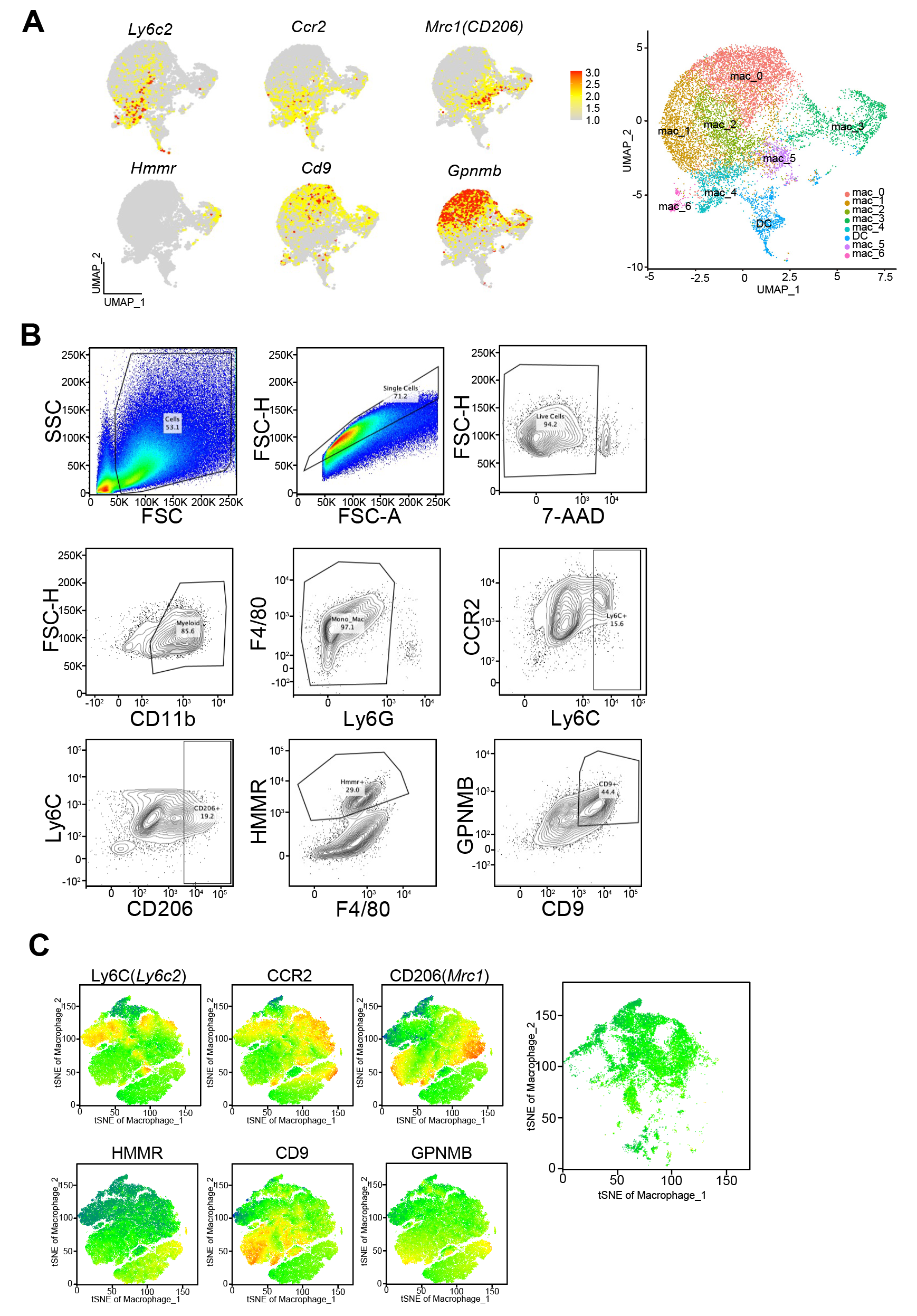
Figure S4, related to Figure 3. Surface antigens that would enable Mac_1 to be fractionated with flow cytometry.**

1. UMAP plots of macrophages projecting normalized expression of major cell surface marker genes in each subpopulation. Right panel shows original UMAP plot with clustering.
2. Strategy for flow cytometric analysis of macrophage subpopulations.
3. tSNE plots of flow cytometry results for the cell surface markers in (A).

**Figure S5, related to Figure 3. Receptors transiently induced in the expanding MuSCs.**

Pseudo-time of gene expression of *Met*, *Cd151*, *Hmmr*, *Rhoa*, *Bsg*, *Cd44*, *Tfrc* and *Grh2*.

**
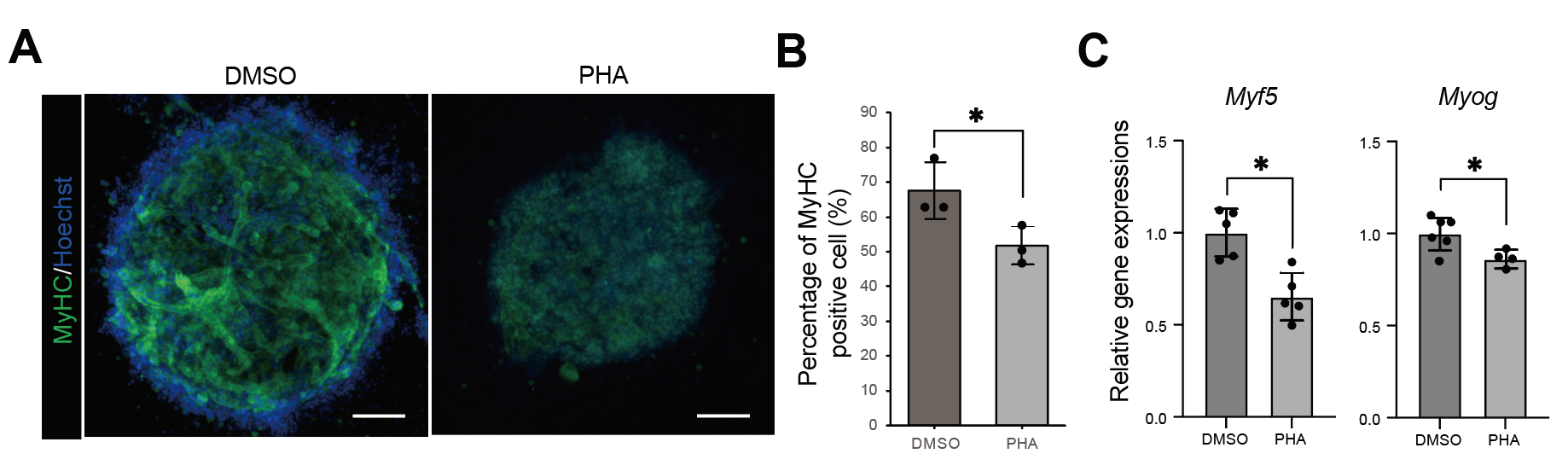
**

**Figure S6, related to Figure 4. HGF/MET signal was responsible for promoting skeletal muscle regeneration in the organoid system.**

1. Staining of MyHC in organoids treated with PHA-665752 (PHA) on day 4 of culture. Scale 100 μm.
2. Quantification of the ratio of MyHC-positive cells (cells within green color areas) in (A). n = 3. Data are expressed as the means ± SD. * *p* < 0.05 (unpaired two-tailed Student’s *t*-test).
3. Gene expression of *Myf5* on day 1 and *Myog* on day 4 in skeletal muscle organoids treated with or without PHA. n = 5 on day1. n = 6 for DMSO and n = 4 for PHA on day4. Data are expressed as the means ± SD. * *p* < 0.05 (unpaired two-tailed Student’s *t*-test).

**
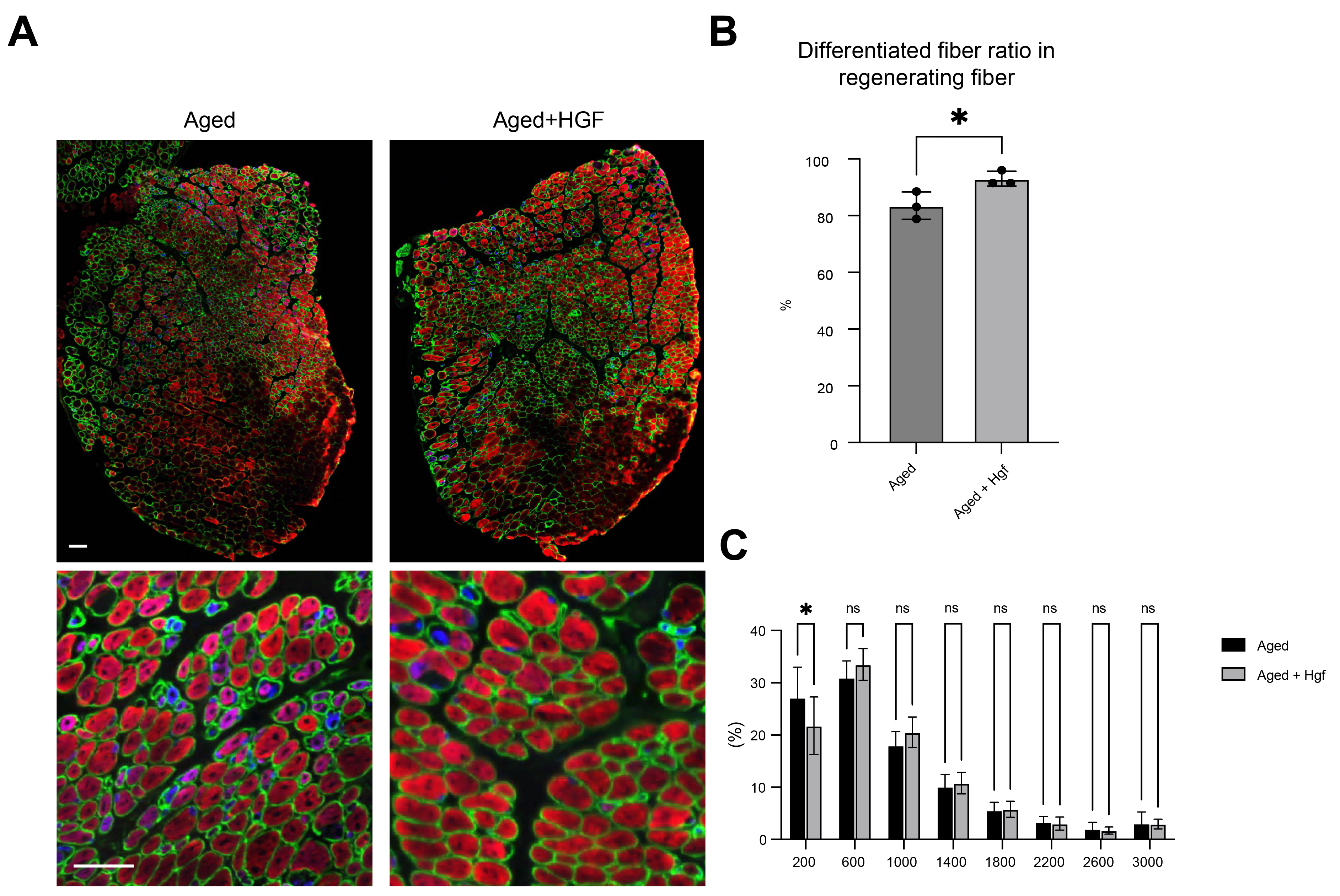
Figure S7, related to Figure 6. The delayed muscle regeneration was observed in older mice.**

1. Representative images of immunofluorescent staining for MYOZ1 and MYH3 in TA sections from aged mice treated with or without HGF on day 7 after CTX administration *in vivo*. Scale: 100 μm.
2. Quantification of the ratio of regenerated MYOZ1-positive fibers among total regenerating fibers shown in image (A). Data are expressed as the means ± SD. n = 3 mice. MYOZ1‑positive regenerated fibers and MYH3-positive regenerating fibers were counted as the total regenerating fiber number. * *p* < 0.05 (unpaired two-tailed Student’s *t*-test).
3. Distribution of muscle fiber cross-sectional areas. The mean number of muscle fibers within the indicated areas per 2,000 myofibers/condition. Data are expressed as the means ± SD. * *p* < 0.05 (one-way ANOVA with Tukey’s multiple comparisons test).
